# The functional effects of *Calanus finmarchicus* hydrolysate as a novel feed ingredient for whiteleg shrimp (*Litopenaeus vannamei*)

**DOI:** 10.3389/fphys.2026.1820807

**Published:** 2026-06-01

**Authors:** Isak Bøgwald, Alice Marie Pedersen, Jorge Dias, Sileshi Gizachew Wubshet, Karl-Erik Eilertsen

**Affiliations:** 1 The Norwegian College of Fishery Science, Faculty of Biosciences, Fisheries, and Economics, UiT The Arctic University of Norway, Tromsø, Norway; 2 Calanus AS, Tromsø, Norway; 3 Norinnova AS, Tromsø, Norway; 4 Sparos Lda, Olhão, Portugal; 5 Nofima AS, Norwegian Institute of Food, Fisheries and Aquaculture, Ås, Norway

**Keywords:** aquaculture, *Calanus finmarchicus*, growth, health, hydrolysate, *Litopenaeus vannamei*, novel ingredient, shrimp

## Abstract

**Introduction:**

Whiteleg shrimp (*Litopenaeus vannamei*) faces nutritional and disease challenges exacerbated by reliance on plant proteins with limited amino acid profiles and antinutritional factors. Functional marine-derived ingredients can improve growth, immunity, and resilience, but sustainable alternatives are needed. Zooplankton, particularly the copepod *Calanus finmarchicus*, represents a vast, underutilized biomass with promising potential as a novel feed resource.

**Methods:**

Five experimental diets were formulated for whiteleg shrimp (*L. vannamei*), including a control and four test diets with 50% reduced fishmeal, compensated by soybean meal and one of four marine ingredients: *C. finmarchicus* hydrolysate (CH), squid-liver meal, krill meal, or tuna hydrolysate. Diets were produced by extrusion and standardized for protein, lipid, and energy content. A 62-day feeding trial was conducted, assessing growth performance, feed utilization, and whole-body composition. Following the trial, shrimp were subjected to an acute salinity challenge to evaluate immune, oxidative stress, and metabolic responses.

**Results:**

Inclusion of CH supported growth comparable to squid-liver and krill meal, with improved feed efficiency and nutrient retention relative to control and tuna hydrolysate diets. CH-fed shrimp showed enhanced protein and energy retention. Following a hyposalinity challenge, survival was highest in shrimp fed CH and krill meal, accompanied by elevated immune activity, reduced lipid peroxidation, and increased antioxidant enzyme responses. Metabolic markers indicated lower amino acid catabolism and improved osmoregulatory adaptation in shrimp fed CH, supporting resilience under environmental stress.

**Discussion:**

This study demonstrates that CH can replace 50% of fishmeal in whiteleg shrimp (*L. vannamei*) diets without compromising growth. CH improved feed efficiency, nutrient retention, and resilience to salinity stress through enhanced antioxidant defenses, immune functionality, and metabolic adaptations. These benefits highlight CH as a sustainable, functional ingredient for shrimp aquaculture. Further research should optimize inclusion levels and validate long-term effects across production systems.

## 1 Introduction

Aquaculture is the fastest-growing food production sector globally, having surpassed capture fisheries in annual production volumes. It provides important nutritional, societal, and economic benefits for over half of the global population (FAO, 2022). While the capacity to produce aquatic animals has increased drastically, the available volume of feed ingredients is lagging. This pertains especially to marine ingredients crucial for optimal farming efficiency of carnivorous fish and crustaceans (Turchini et al., 2019). Whiteleg shrimp (*Litopenaeus vannamei*) is central to global aquaculture production and accounted for 51.7% of the global crustacean production in 2020 (FAO, 2022). Although being a commercial success, shrimp aquaculture still faces significant challenges, such as disease outbreaks and environmental stressors, that threaten both animal welfare and economic sustainability (Asche et al., 2021). These challenges are particularly evident in pond-based farming systems, where sudden environmental changes such as heavy rainfalls may lead to increased disease susceptibility and mortality due to abrupt drops in water salinity (Van Thuong et al., 2016).

Ensuring optimal nutrition through improved feed formulations is critical for enhancing shrimp health and survival. The limited availability and high cost of traditional marine-based protein sources, such as fishmeal, have forced the industry to rely heavily on plant proteins for diet production. Even though plant-based ingredients like soybeans are considered economically and nutritionally viable, they have notable disadvantages compared to marine-derived ingredients. Plant proteins have lower levels of essential amino acids, the presence of antinutrients and mycotoxins, and lower attractivity and palatability for marine crustaceans (Oliveira and Vasconcelos, 2020; Yuan et al., 2021; Chen et al., 2024). To meet these challenges, functional ingredients rich in bioactive marine compounds are incorporated into plant-based diets. Such functional ingredients improve growth, immunity, and stress tolerance, compensating for the nutritional and performance deficits observed with plant-based diets (Hossain et al., 2024). However, the large volumes of feed required for farming species such as whiteleg shrimp shed light on the demand for novel sustainable protein sources to support further increases in shrimp production capacity and efficiency.

Zooplankton is a diverse group of low-trophic organisms with considerable potential as new feed ingredients for aquaculture. Their position in the marine ecosystem and their role as natural starter feed for many aquatic animals emphasize an optimal nutrient composition, and they represent a vast biomass currently underutilized for food and feed applications. Utilizing zooplankton and other low-trophic organisms according to the proposed “balanced harvesting” approach has been suggested to reduce the ecological impact of fisheries while increasing overall fishery yield (Zhou et al., 2019). Antarctic krill (*Euphausia superba*) is one of the species that has been harvested for decades, and the inclusion of krill meal in diets for whiteleg shrimp has been shown to benefit palatability, growth, and immunity (Nunes et al., 2019; Wei et al., 2022). Another promising candidate is the marine copepod *Calanus finmarchicus*, which is abundant in the Arctic and subarctic waters, with an estimated annual production of approximately 290 million tonnes in the Norwegian Sea and adjacent regions (Skjoldal, 2004; Broms et al., 2016). Despite its high biomass availability, optimal nutrient profile, together with potential for sustainable harvesting, the use of *C. finmarchicus* in aquaculture remains largely unexplored. Recent developments in harvesting techniques and precautionary governmental management plans have made *C. finmarchicus* a viable resource for aquaculture (Albrektsen et al., 2022; Fjeld et al., 2023).

A novel protein hydrolysate derived from C. finmarchicus has been shown to increase feed attractiveness for whiteleg shrimp (Bøgwald et al., 2024). However, its effects on growth performance, health, and resilience under environmental stress have not been investigated. Addressing these effects is critical to harness the potential of *C. finmarchicus* as a sustainable and functional feed ingredient for shrimp aquaculture. The objectives of the current study were to use a feeding trial to benchmark the hydrolysate against other similar ingredients for growth and health, and to evaluate its ability to compensate for a 50% reduction in dietary fishmeal. The aim was to study the efficacy of *C. finmarchicus* hydrolysate in diets for whiteleg shrimp, ultimately to provide the shrimp industry with a novel functional feed ingredient. We hypothesized that dietary inclusion of CH would compensate for a 50% reduction in fishmeal by maintaining or improving growth performance and feed efficiency, and that CH-fed shrimp would exhibit enhanced immune and antioxidant responses under hypo salinity stress, compared with a standard fishmeal-containing control diet.

## 2 Methods

### 2.1 Experimental diets

#### 2.1.1 Test ingredients

*Calanus finmarchicus* raw material was harvested from the Norwegian Sea by Calanus AS (Tromsø, Norway) using patented trawl technology. The harvested biomass was processed into calanus hydrolysate (CH) through enzymatic hydrolysis of the whole organism, followed by concentration and stabilization of the resulting liquid fraction, a process that preserves the free amino acids, bioactive peptides, and lipid components of the intact organism (Albrektsen et al., 2022). The three commercial marine ingredients of squid-liver meal (SLM), krill meal (KM), and tuna hydrolysate (TH), were supplied by SPAROS Lda (Olhão, Portugal). CH and TH were in liquid form and SLM and KM were dry powders. The nutrient composition of the test ingredients is presented in **Table 1**.

**Table 1:** Nutrient composition of test ingredients.

Product form	Liquid	Powder	Powder	Liquid
Test ingredient	CH	SLM	KM	TH
Moisture, %	46.7	5.3	5.7	62.1
Crude protein, % DW	63.0	82.5	60.1	61.2
Crude fat, % DW	1.1	10.3	18.6	7.9
Ash, % DW	20.5	9.0	12.3	18.7
Phosphorus, % DW	1.48	1.3	1.8	5.0
Gross energy, kJ/g DW	18.4	23.3	23.1	19.0
Arginine, %	2.23	4.15	3.20	1.14
Histidine, %	0.46	0.96	1.26	0.85
Isoleucine, %	1.21	1.44	2.93	0.59
Leucine, %	2.07	3.16	4.37	1.09
Lysine, %	2.26	3.58	3.78	1.16
Threonine, %	1.23	1.93	2.64	0.72
Tryptophan, %	n.a.	0.24	0.75	0.15
Valine, %	1.58	2.28	3.00	0.77
Methionine, %	0.62	0.79	1.66	0.40
Cysteine, %	0.33	0.47	0.54	0.14
Phenylalanine, %	1.08	1.86	2.79	0.59
Tyrosine, %	1.19	0.90	2.31	0.47
Aspartic acid, %	2.56	5.07	5.90	1.37
Glutamic acid, %	3.85	9.05	7.22	2.12
Alanine, %	2.12	5.40	3.05	1.34
Glycine, %	2.29	9.81	2.67	1.91
Proline, %	1.13	6.13	3.07	1.09
Serine, %	1.11	2.44	2.32	0.74

n.a., not available; DW, Dry weight; CH, Calanus hydrolysate; SLM, Squid-liver meal; KM, Krill meal; TH, Tuna hydrolysate.

#### 2.1.2 Formulation of experimental diets

The trial comprised five experimental diets (**Table 2**). A control diet (CTRL) was made to mimic a standard formulation for whiteleg shrimp. In comparison, the other four diets were formulated with a 50% reduction of fishmeal, compensated by the inclusion of a test ingredient and a higher level of soybean meal. The inclusion level of calanus hydrolysate was set at 5%, and the inclusion levels of the other test ingredients were adjusted by their crude protein content (**Table 1**) to allow equivalent protein supply for each diet. Diets were formulated as isonitrogenous, isolipidic and isoenergetic.

**Table 2:** Formulation of experimental diets.

Ingredients, %	CTRL	CH	SLM	KM	TH
Fishmeal ^1^	15.00	7.50	7.50	7.50	7.50
Calanus hydrolysate ^2^		5.00			
Squid-liver meal ^2^			3.80		
Krill meal ^2^				5.20	
Tuna hydrolysate ^2^					5.10
Poultry meal ^3^	5.00	5.00	5.00	5.00	5.00
Wheat gluten ^4^	2.20	2.20	2.20	2.20	2.20
Soybean meal 44 ^5^	28.00	32.53	32.00	32.20	32.50
Wheat meal ^6^	30.83	28.10	30.13	29.13	28.33
Rice bran full fat ^7^	10.00	10.00	10.00	10.00	10.00
Fish oil ^8^	1.00	1.00	1.00	1.00	1.00
Soybean oil ^9^	1.90	2.60	2.30	1.70	2.30
Soy lecithin ^10^	0.50	0.50	0.50	0.50	0.50
Cholesterol SF ^11^	0.12	0.12	0.12	0.12	0.12
Vitamin and mineral premix ^12^	1.00	1.00	1.00	1.00	1.00
Dicalcium phosphate ^13^	1.80	1.80	1.80	1.80	1.80
Calcium carbonate ^14^	1.10	1.10	1.10	1.10	1.10
Guar gum ^15^	1.00	1.00	1.00	1.00	1.00
L-Lysine HCl 99% ^16^	0.40	0.40	0.40	0.40	0.40
DL-Methionine ^17^	0.15	0.15	0.15	0.15	0.15

^1^ CONRESA 60: 61% CP, 11% CF, Conserveros Reunidos S.A., Spain; ^2^Details in **Table 1**; ^3^Poultry meal 65: 65% CP, 12% CF, SAVINOR UTS, Portugal; ^4^VITEN: 81% CP, 2.1% CF, Roquette, France; ^5^Solvent extracted soybean meal: 43% CP, 2.7% CF, CARGILL, Spain; ^6^Wheat meal: 10.2% CP; 1.2% CF, MOLISUR, Spain; ^7^Rice bran (full fat): 13.3% CP; 16.3% CF, Ribeiro e Sousa Lda, Portugal; ^8^Sopropêche, France; ^9^J.C. Coimbra Lda, Portugal; ^10^LECICO GmbH, Germany; ^11^CARBOGEN AMCIS B.V, The Netherlands; ^12^PREMIX Lda, Portugal: Vitamins (IU or mg/kg diet): DL-alpha tocopherol acetate, 100mg; sodium menadione bisulphate, 25mg; retinyl acetate, 20, 000 IU; DL-cholecalciferol, 2, 000 IU; thiamin, 30mg; riboflavin, 30mg; pyridoxine, 20mg; cyanocobalamin, 0.1mg; nicotinic acid, 200mg; folic acid, 15mg; ascorbic acid, 500mg; inositol, 500mg; biotin, 3mg; calcium pantothenate, 100mg; choline chloride, 1, 000mg, betaine, 500mg, excipient wheat; ^13^DCP: 16.8% P, 20.9% Ca, PREMIX Lda, Portugal; ^14^Calcium carbonate: 40% Ca, PREMIX Lda, Portugal; ^15^Seah International, France; ^16^L-Lysine HCl 99%: Ajinomoto Eurolysine SAS, France; ^17^Rhodimet NP99, ADISSEO, France. CTRL, Control; CH, Calanus hydrolysate; SLM, Squid-liver meal; KM, Krill meal; TH, Tuna hydrolysate.

#### 2.1.3 Production of experimental diets

The diets were produced by extrusion at the SPAROS facilities. All powder and liquid hydrolysates were mixed in a double-helix mixer (model 500L, TGC Extrusion, France) and ground below 400 µm in a micropulverizer hammer mill (model SH1, Hosokawa-Alpine, Germany). Pellets with sizes of 1.2 mm and 2.0 mm were manufactured using a twin-screw extruder (model BC45, Clextral, France) with a screw diameter of 55.5 mm. Conditions during extrusion were: Feeder rate 50–54 kg/h, screw speed 175–185 rpm, water addition 365 ml/min, temperature barrel 1 32-34°C, temperature barrel 3 104-107°C. Extruded pellets were dried in a vibrating fluid bed dryer (model DR100, TGC Extrusion, France). Oils were added after cooling by top-coating (model PG-10VCLAB, Dinnissen, The Netherlands). Diets were packed in sealed plastic buckets and stored at room temperature throughout the trial. Samples of each diet were taken for compositional analyses, presented in **Table 3** (detailed analytics in **Supplementary Table 1** in **Supplementary Data**).

**Table 3:** Analytical composition of experimental diets.

Parameter	CTRL	CH	SLM	KM	TH
Moisture, %	6.66 ± 0.02	6.54 ± 0.02	6.41 ± 0.02	6.19 ± 0.03	6.62 ± 0.03
Crude protein, %	32.66 ± 0.03	32.73 ± 0.15	32.74 ± 0.05	32.73 ± 0.08	32.70 ± 0.05
Crude fat, %	7.12 ± 0.17	7.12 ± 0.15	7.08 ± 0.08	7.15 ± 0.07	7.19 ± 0.05
Ash, %	9.15 ± 0.04	8.03 ± 0.01	8.20 ± 0.06	8.64 ± 0.00	8.48 ± 0.04
Phosphorus, %	1.03 ± 0.01	0.94 ± 0.01	1.01 ± 0.03	1.02 ± 0.05	0.93 ± 0.01
Gross energy, kJ/g	18.36 ± 0.03	18.37 ± 0.09	18.35 ± 0.08	18.43 ± 0.09	18.48 ± 0.03
Arginine, %	2.09 ± 0.02	2.01 ± 0.02	2.06 ± 0.02	2.09 ± 0.01	2.01 ± 0.01
Histidine, %	0.78 ± 0.01	0.75 ± 0.02	0.81 ± 0.01	0.78 ± 0.01	0.73 ± 0.01
Isoleucine, %	1.27 ± 0.01	1.29 ± 0.01	1.29 ± 0.02	1.33 ± 0.03	1.23 ± 0.03
Leucine, %	2.34 ± 0.03	2.33 ± 0.01	2.34 ± 0.03	2.37 ± 0.02	2.27 ± 0.01
Lysine, %	2.20 ± 0.02	2.15 ± 0.03	2.14 ± 0.03	2.19 ± 0.03	2.12 ± 0.02
Threonine, %	1.29 ± 0.03	1.22 ± 0.03	1.25 ± 0.03	1.28 ± 0.02	1.24 ± 0.02
Tryptophan, %	0.42 ± 0.01	0.42 ± 0.01	0.41 ± 0.00	0.41 ± 0.01	0.40 ± 0.01
Valine, %	1.55 ± 0.04	1.52 ± 0.02	1.53 ± 0.01	1.50 ± 0.02	1.45 ± 0.01
Methionine, %	0.72 ± 0.02	0.72 ± 0.01	0.75 ± 0.01	0.73 ± 0.01	0.68 ± 0.01
Cysteine, %	0.48 ± 0.01	0.51 ± 0.01	0.50 ± 0.01	0.48 ± 0.01	0.47 ± 0.02
Phenylalanine, %	1.44 ± 0.02	1.48 ± 0.01	1.51 ± 0.01	1.56 ± 0.01	1.43 ± 0.01
Tyrosine, %	1.05 ± 0.02	1.03 ± 0.03	1.13 ± 0.03	1.05 ± 0.01	1.07 ± 0.01
Aspartic acid, %	2.93 ± 0.05	2.91 ± 0.03	3.08 ± 0.03	3.06 ± 0.05	2.96 ± 0.02
Glutamic acid, %	6.19 ± 0.05	6.29 ± 0.03	6.18 ± 0.02	6.31 ± 0.04	6.10 ± 0.03
Alanine, %	1.68 ± 0.03	1.69 ± 0.03	1.61 ± 0.03	1.63 ± 0.02	1.58 ± 0.02
Glycine, %	2.03 ± 0.03	2.16 ± 0.02	1.87 ± 0.03	1.96 ± 0.01	1.85 ± 0.02
Proline, %	2.03 ± 0.03	2.15 ± 0.01	1.94 ± 0.03	2.08 ± 0.04	2.07 ± 0.01
Serine, %	1.60 ± 0.04	1.58 ± 0.02	1.59 ± 0.03	1.60 ± 0.01	1.55 ± 0.03

Values are means ± standard deviation (*n* = 2). CTRL, Control; CH, Calanus hydrolysate; SLM, Squid-liver meal; KM, Krill meal; TH, Tuna hydrolysate.

### 2.2 Experimental trial

#### 2.2.1 Ethical statement

The trial was conducted under the supervision of SPAROS Lda, at the experimental facilities of RIASEARCH (Murtosa, Portugal), which are registered for experimentation with aquatic species (015609/2017) by Direção-Geral de Alimentação e Veterinária (Ministry of Agriculture, Portugal). The experimental protocol was approved by the Animal Welfare Committee - Órgão Responsável pelo Bem-Estar Animal (ORBEA) (study ref. CALSHRIMP2101). Experiments were conducted by FELASA certified scientists and technical staff, in full compliance with ARRIVE guidelines and the European (Directive 2010/63/EU) and Portuguese (Decreto-Lei n°. 113/2013, August 7th) legislation on the protection of animals for scientific purposes.

#### 2.2.2 Shrimp

The experimental species under testing was whiteleg shrimp (Litopenaeus vannamei), originating from Shrimp Improvement Systems LLC (Florida, USA). A stock of post-larvae shrimp (± 30 000) were transferred to the experimental facilities by an authorized carrier and kept on sanitary quarantine for 2 weeks. No pathological signs were observed in association to transport. Prior to the start of the trial, the shrimp stock was kept in four 500 L tanks supplied with recirculated seawater (salinity 20 ‰, water-flow 2.3 L/min, temperature 26 ± 1°C, dissolved oxygen kept above 6 mg/L) for 2 months. During this period, the shrimp were fed a commercial diet (WinFlat, SPAROS Lda, Portugal) with automatic feeders at approximately 12% biomass per day. The shrimp were manually sorted prior to the start of the trial to constitute a sub-stock with a homogenous weight range.

#### 2.2.3 Growth performance trial

A total of 2,000 shrimp were distributed into 25 tanks (80 shrimp per tank), with five tanks for each of the five experimental diets. The mean initial body weight of the shrimp was 2.15 ± 0.34 g, and they were fed the experimental diets for 62 days. The shrimp were grown in 500L circular tanks supplied with recirculated seawater, and they were subjected to photoperiod conditions of 12 hours light and 12 hours dark. During the growth performance trial, the average water temperature was 28.4 ± 0.3°C, salinity was 20.0 ± 0.4 ppt, and dissolved oxygen levels were kept above 5.9 mg/L (details in **Supplementary Table 2** in **Supplementary Data**). The shrimp were manually fed in five daily meals at 08:00, 12:00, 14:00, 16:00, and 19:00. Daily feed rations were based on a commercial feeding table for shrimp. However, the feed ration per tank was adjusted daily to avoid feed wastage. The feed rations were increased by 0.2% if all pellets were eaten, maintained if less than 5 pellets remained in the tank, and decreased by 0.2% if more than 5 pellets remained in the tank.

The shrimp were group-weighed after 62 days of feeding, and the total biomass divided by the number of animals resulted in the final body weight. A pool of 20 shrimp from the initial stock at the start and 10 shrimp per tank at the end of the trial were sampled for analysis of whole-body composition (moisture, ash, protein, fat, and energy content). Growth performance criteria were calculated using these formulas:

Specific growth rate, SGR(%/day):(Ln FBW–Ln IBW)×100/days

Feed conversion ratio, FCR: crude feed intake/weight gain

Protein efficiency ratio, PER: wet weight gain/crude protein intake

$\text{Retention }(\%)=100\text{ x }\frac{\left(\text{FBW x NFF})-(\text{IBW x NIF}\right)}{\text{Nutrient intake}}$

Feed intake, FI(%BW/day):(crude feed intake/(IBW+FBW)/2/days)×100

IBW (g): Initial mean body weight. FBW (g): Final mean body weight. NFF: Nutrient content of final shrimp. NIF: Nutrient content of initial shrimp.

#### 2.2.4 Salinity challenge

After completing the growth experiments, all (shrimp/tank/diet) remaining/live shrimp groups/tanks were subjected to an acute environmental stress challenge, reducing the water salinity from 20 ppt to 5 ppt within a period of 24 hours. The salinity challenge was performed using the same tanks with the remaining shrimp/groups completing the growth experiment under the same conditions (except for salinity). Seven days after this salinity drop, 2 shrimp per replicate tank (10 shrimp per dietary treatment) were sampled for collection of hemolymphs, withdrawn from the ventral sinus into a 1 ml disposable syringe containing 1:1 proportion of an anticoagulant buffer (27 mM sodium citrate, 385 mM NaCl, 115 mM glucose; pH 7.5). The hepatopancreas of the same shrimp (10 per dietary treatment) was also collected. All the samples were frozen in liquid nitrogen and stored at -80°C until analysis of immune biomarkers, oxidative stress, and metabolic status.

### 2.3 Analytical methods

#### 2.3.1 Biochemical compositions of diets and whole shrimp

Analysis of test ingredients, experimental diets, and whole shrimp were performed with analytical duplicates and following the methodology described by AOAC (2006): Dry matter was determined after drying at 105°C for 24 h; ash by combustion at 550°C for 6 h in a muffle furnace (Nabertherm L9/11/B170, Germany); crude protein (N x 6.25) by a flash combustion technique followed by a gas chromatographic separation and thermal conductivity detection with a Leco N Analyzer (Model FP-528, Leco Corporation, USA); crude lipid by petroleum ether extraction at 40-60°C using a Soxtec™ 2055 Fat Extraction System (Foss, Denmark) with prior acid hydrolysis with 8.3 M HCl; gross energy in an adiabatic bomb calorimeter (Werke C2000, IKA, Germany). Total phosphorus was analyzed at an external laboratory (Eurofins Anàlisis Alimentario SL, Spain) according to the ISO 27085:2009 by ICP-AES methodology (ISO, 2009). Amino acids in test ingredients and diets were determined after hydrolysis in 6M HCL at 108°C for 24 h, using a Waters Pico-Tag reversed-phase HPLC system with norleucine as internal standard.

#### 2.3.2 Hemolymph immune biomarkers

Lysozyme level was assessed using a turbidimetric assay following the methodology described Costas et al. (2011). Prophenoloxidase (ProPo) activity was determined photometrically. Plasma samples were diluted in TBS (10 mM Tris, 336 mM NaCl, 5 mM CaCl_2_, 10 mM MgCl_2_, pH 7.6) and pre-incubated with an equal volume of the enzyme inducer trypsin (Sigma-Aldrich). Trypsin and plasma were replaced by TBS. After incubation, the red pigment L-DOPA was added and the formation of DOPA-chrome was monitored after 0, 5, 15, and 25 minutes. ProPo activity was measured as the variation in absorbance (490 nm) per minute and the level was expressed in U/mg protein. The bactericidal capacity (*Vibrio* inhibitory activity) was measured according to the method of Graham et al. (1988), with some modifications by MaChado et al. (2015). Briefly, 10 µl plasma was added to the wells of a microplate, using Hanks’ Balanced Salt Solution (HBSS) instead of plasma for the positive control. Each well received 20 µl of *Vibrio harveyi* (3.13 x 10^6^ CFU/ml) followed by an incubation for 2.5 h at 25°C. After incubation, 25 µl of 3-(4, 5 dimethyl-2-yl)-2, 5-diphenyl tetrazolium bromide (1 mg/ml, Sigma-Aldrich) was added to each well before another incubation at 25°C for 10 min. The microplates were then centrifuged at 2000 x g for 10 min and the precipitate was dissolved in 200 µl of dimethyl sulfoxide. Absorbance of dissolved formazan was read at 540 nm. The bactericidal capacity was expressed as the percentage of killed bacteria, calculated as the difference between absorbance of surviving bacteria compared to the absorbance of bacteria from positive controls (100%).

#### 2.3.3 Hepatopancreas oxidative stress and metabolic enzymes

The hepatopancreas samples were homogenized prior to the analyses of oxidative stress biomarkers and antioxidative enzymes. The soluble protein concentration of the hepatopancreas samples was determined using the Micro BCA™ Protein Assay Kit (Thermo Scientific, Ref. 23235), with bovine serum albumin as a standard. Protein carbonyls (PC) content in homogenized hepatopancreas samples was determined using a commercial kit (Sigma-Aldrich, Ref. MAK094). Superoxide dismutase (SOD) activity was measured by the ferricytochrome C method according to McCord and Fridovich (1969). Catalase (CAT) activity was determined by measuring the decrease in hydrogen peroxide concentration according to Aebi (1984). Total glutathione (tGSH) was measured following the method described by Griffith (1980) and Vandeputte et al. (1994) with modifications. Analysis of tGSH was performed at 37°C, and absorbance changes due to reduction of 5, 5’-dithiobis-(2-nitrobenzoic acid) (DTNB, Sigma-Aldrich) were monitored at 412 nm in a Multiskan GO microplate reader. The molar extinction coefficient used for DTNB was 13–600 M^-1^ x cm^-1^. tGSH was determined using a reaction mixture containing 133 mM phosphate buffer with 5.8 mM EDTA at pH 7.4, 0.71 mM DTNB, 0.24 mM NADPH, and 1.2 IU/ml GR. Lipid peroxidation (LPO) was determined by assessing the concentration of thiobarbituric acid reactive substances (TBARS) according to the method of Buege and Aust (1978). Alanine aminotransferase activity (ALT) and aspartate aminotransferase (AST) were determined using commercial kits, GPT/ALT Kit (Spinreact, Ref. 41280) and GOT/AST Kit (Spinreact, Ref. 41273).

#### 2.3.4 Free amino acids and nitrogen metabolites

For the quantification of free amino acids and nitrogen-related metabolites, the hepatopancreas samples were homogenized (0.1 M HCl on ice) and centrifuged (1500g, 15min, 4°C). The supernatant was deproteinized by centrifugal ultrafiltration (10 kDa cut-off, 2500 x g, 20 min, 4°C). The samples were then pre-column derivatized with Waters AccQ Fluor Reagent (6-aminoquinolyl-N-hydroxysuccinimidyl carbamate) using the AccQ Tag method (Waters, USA). Analyses were done by ultra-high-performance liquid chromatography (UHPLC) in a Waters reversed-phase amino acid analysis system with norvaline as internal standard. The resulting peaks were analyzed with the EMPOWER software (Waters, USA).

### 2.4 Statistical analysis

All data were subjected to a one-way analysis of variance (ANOVA) after assessing normality using the Shapiro-Wilk’s test. Values expressed as percentages were subjected to arcsine square root transformation prior to ANOVA. Variance homogeneity was confirmed with the Brown-Forsythe test. Differences between the means were assessed with the Tukey’s *post hoc* test (**Supplementary Table 10** in the **Supplementary Data**). Statistical significance was tested at a 0.05 probability level. All statistical tests were performed using GraphPad Prism (version 10.2.0). Results were presented either in tables, as bars with means ± standard deviation, or as box (25^th^ to 75^th^ percentiles with line at median) and whiskers (min to max) plots with *n* = 5. Color schemes were made using the “colorblind safe” option with ColorBrewer 2.0 (Harrower and Brewer, 2003).

## 3 Results

**Figure 1:**
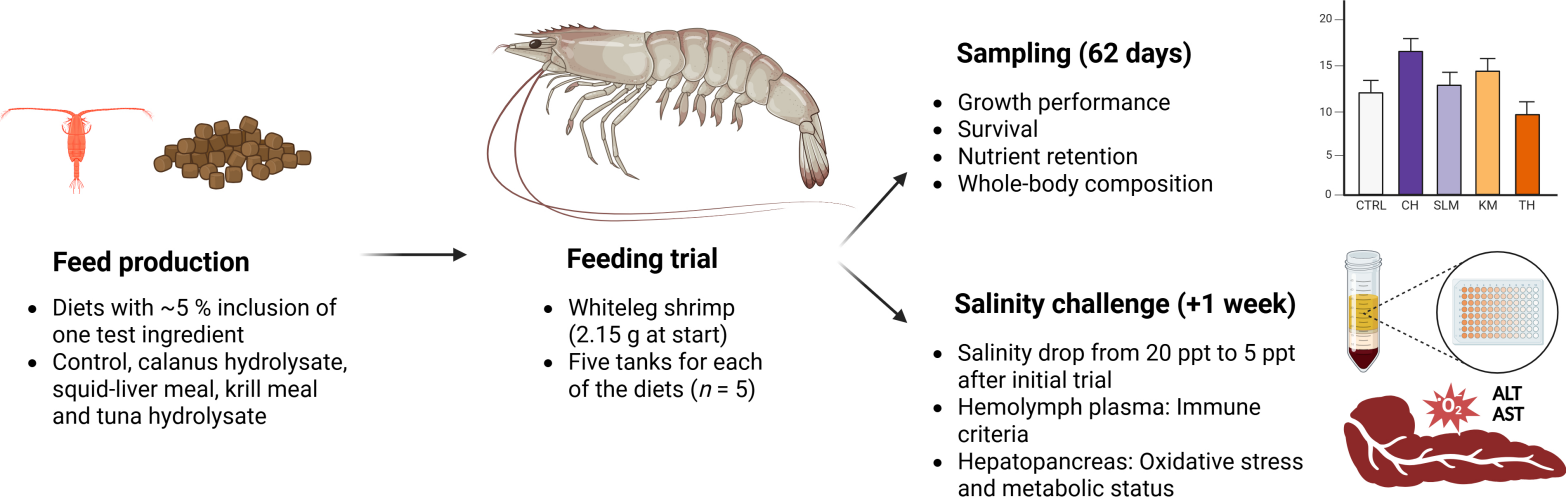
Experimental workflow of the feeding trial assessing the effects of *C. finmarchicus* hydrolysate and other functional ingredients in diets for whiteleg shrimp. CTRL, Control; CH, Calanus hydrolysate; SLM, Squid-liver meal; KM, Krill meal; TH, Tuna hydrolysate; ALT, Alanine aminotransferase; AST, Aspartate aminotransferase. Figure created with BioRender.com.

Whiteleg shrimp readily accepted all the experimental diets from the start of the trial (initial weight: 2.15 g) through to its conclusion after 62 days. Following the initial growth trial and associated samplings, salinity was reduced from 20 ppt to 5 ppt to assess the resilience of the shrimp to abrupt environmental stress. Accordingly, results are presented in two parts: (1) performance parameters from the initial trial (growth, body composition, nutrient retention), and (2) outcomes related to health following the salinity challenge (survival, immune response, oxidative stress, and metabolic status). The experimental workflow is illustrated in **Figure 1**, and detailed data for all parameters are found in **Supplementary Tables 3–S9** in the **Supplementary Material**.

### 3.1 Initial trial

#### 3.1.1 Growth performance

**Figure 2:**
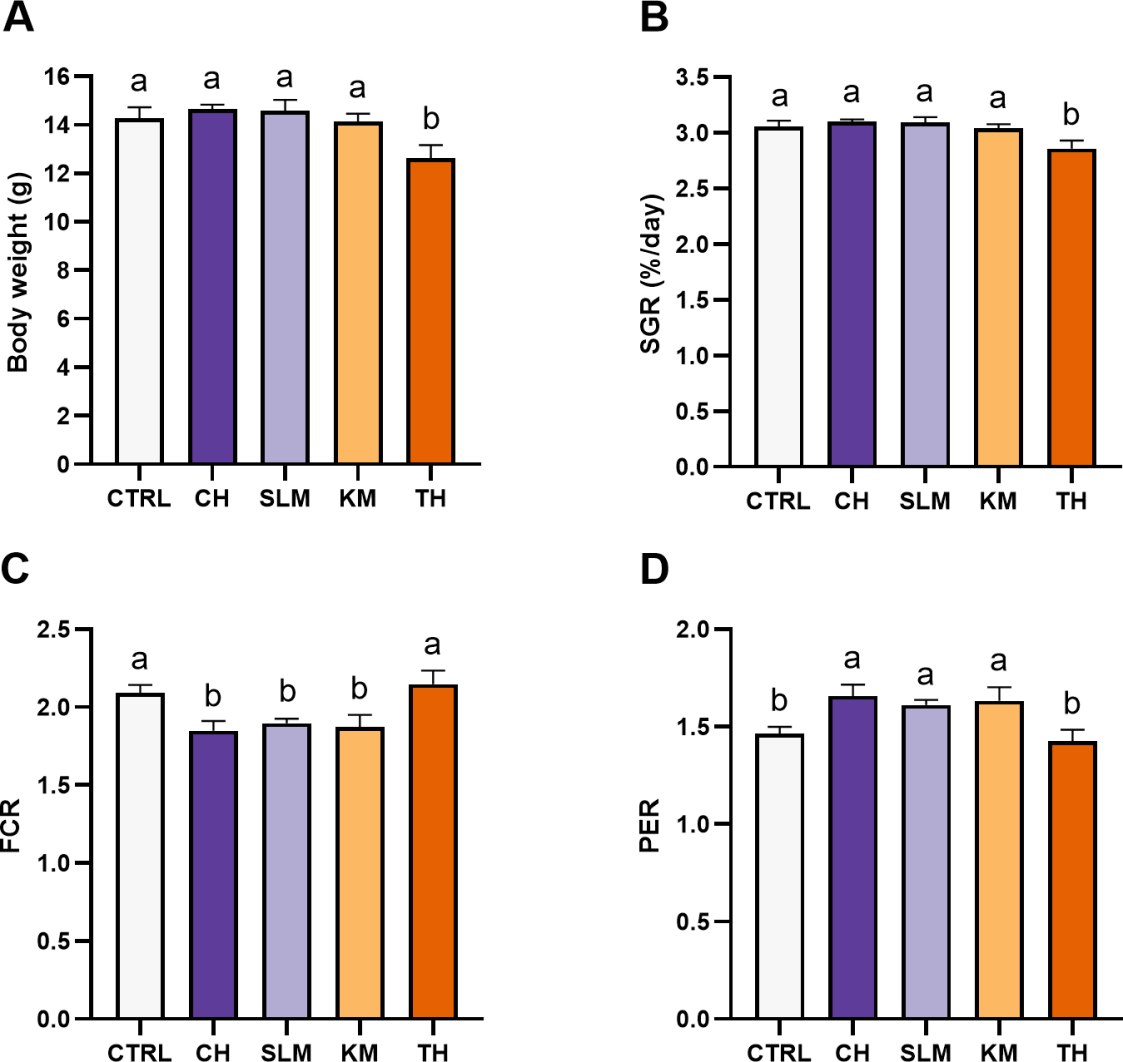
Growth performance of whiteleg shrimp fed the experimental diets for 62 days. **(A)** Final body weight. **(B)** Specific growth rate (SGR). **(C)** Feed conversion ratio (FCR). **(D)** Protein efficiency ratio (PER). CTRL, Control; CH, Calanus hydrolysate; SLM, Squid-liver meal; KM, Krill meal; TH, Tuna hydrolysate. Bars are means ± standard deviations (*n* = 5). Different lower-case letters (a–c) indicate statistically significant differences (*p* < 0.05).

Shrimp fed the CH diet showed a 6.8-fold increase in body weight throughout the feeding experiment, which was comparable to shrimp fed diets with SLM and KM but significantly higher than those fed the TH diet (**Figure 2A**). The specific growth rate (SGR) was similar among shrimp fed the CH, SLM, KM, and CTRL diets, whereas shrimp fed the TH diet showed a significantly lower SGR (**Figure 2B**). Feed efficiency, as indicated by FCR and PER, was significantly improved in shrimp fed the CH, SLM, and KM diets, compared to the CTRL and TH diets (**Figures 2C, D**). For example, shrimp fed the CH diet exhibited a 12% improvement in FCR compared to the control group (1.85 vs 2.09, respectively). In addition to growth performance, the nutrient retention and whole-body composition of shrimp fed the experimental diets were analyzed to further evaluate the effects of dietary CH inclusion.

#### 3.1.2 Nutrient retention and whole-body composition

**Figure 3:**
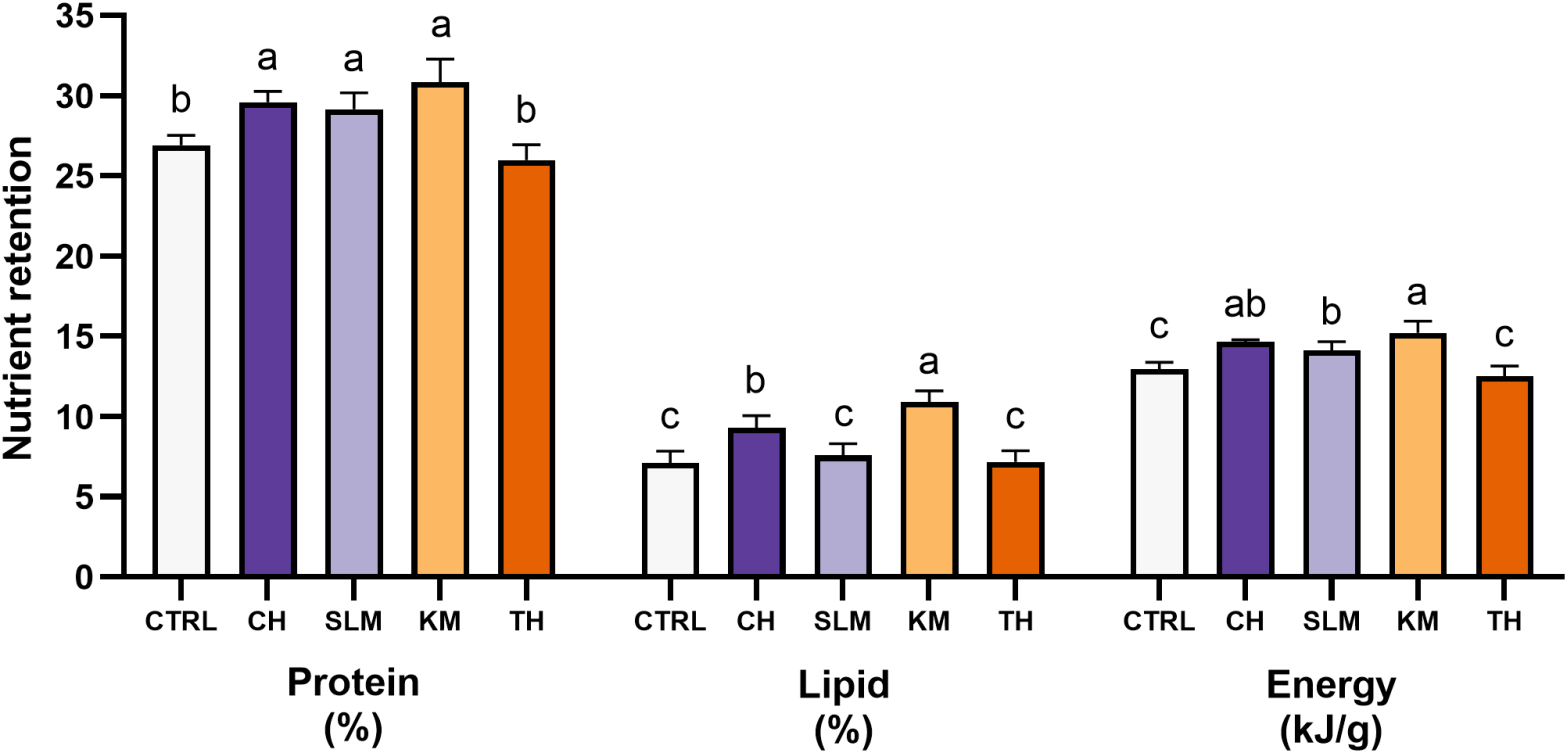
Nutrient retention in whiteleg shrimp after 62 days of feeding. Protein and lipids are presented in % of intake, and energy as kJ/g. CTRL, Control; CH, Calanus hydrolysate; SLM, Squid-liver meal; KM, Krill meal; TH, Tuna hydrolysate. Bars are means ± standard deviations (*n* = 5). Different lower-case letters (a–c) indicate statistically significant differences (*p* < 0.05).

Nutrient retention and whole-body composition were assessed to determine the efficiency of nutrient utilization in shrimp fed the experimental diets. Protein retention was significantly higher in shrimp fed CH, SLM, and KM compared to CTRL and TH diets (**Figure 3**). Lipid and energy retention were highest in shrimp fed KM, followed by CH, both of which significantly outperformed the other diets. More specifically, shrimp fed the CH diet had significantly higher whole-body energy retention (14.7%) compared to the CTRL diet (12.9%), and lipid retention was also significantly improved in shrimp fed the CH diet *(9.3%)* compared to the CTRL diet (7.1%).

Whole-body composition analysis revealed that shrimp fed the KM diet had the highest protein content (17.7%), significantly exceeding that of shrimp fed the CH and TH diets (16.9% and 17.0%, respectively) (**Table 4**). Shrimp fed the KM diet also had the highest fat content (1.3%), while shrimp fed the TH diet exhibited the lowest total energy content (4.6 kJ/g). Shrimp fed the TH diet had significantly higher moisture than those fed KM, while ash content was significantly higher in shrimp fed SLM compared to CTRL.

**Table 4:** Whole-body composition of shrimp after 62 days of feeding.

Parameter	CTRL	CH	SLM	KM	TH
Moisture (%)	76.2 ± 0.2 ^ab^	76.5 ± 0.5 ^ab^	76.3 ± 0.7 ^ab^	75.6 ± 0.4 ^b^	76.7 ± 0.6 ^a^
Ash (%)	2.9 ± 0.1 ^b^	3.0 ± 0.1 ^ab^	3.1 ± 0.1 ^a^	2.9 ± 0.2 ^ab^	3.0 ± 0.1 ^ab^
Protein (%)	17.3 ± 0.1 ^ab^	16.9 ± 0.2 ^b^	17.1 ± 0.5 ^ab^	17.7 ± 0.3 ^a^	17.0 ± 0.4 ^b^
Fat (%)	1.0 ± 0.1 ^bc^	1.1 ± 0.1 ^b^	0.9 ± 0.1 ^c^	1.3 ± 0.1 ^a^	1.0 ± 0.1 ^bc^
Energy (kJ/g)	4.7 ± 0.1 ^ab^	4.7 ± 0.1 ^ab^	4.6 ± 0.2 ^ab^	4.9 ± 0.1 ^a^	4.6 ± 0.2 ^b^

CTRL, Control; CH, Calanus hydrolysate; SLM, Squid-liver meal; KM, Krill meal; TH, Tuna hydrolysate. Values are means ± standard deviations (*n* = 5). Different lower-case letters (a–c) indicate statistically significant differences (*p* < 0.05, *n* = 5).

### 3.2 Hyposalinity challenge

#### 3.2.1 Stress resilience and survival

**Figure 4:**
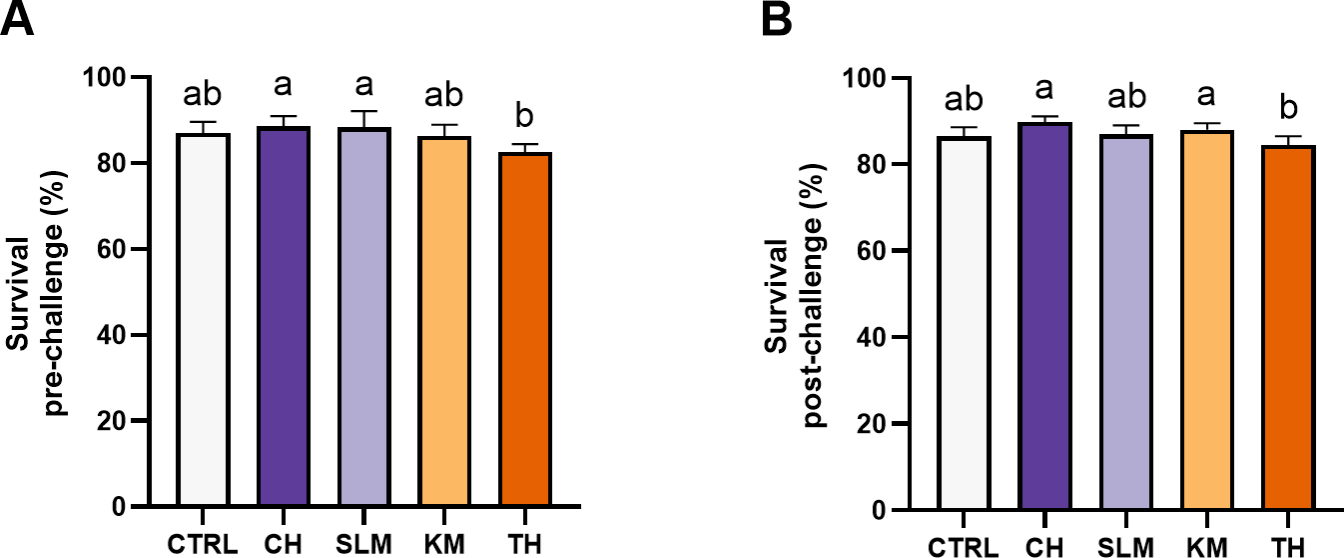
Survival rate of whiteleg shrimp. **(A)** After 62-day initial feeding trial. **(B)** Seven days post-salinity challenge (20 ppt to 5 ppt over 24 h). CTRL, Control; CH, Calanus hydrolysate; SLM, Squid-liver meal; KM, Krill meal; TH, Tuna hydrolysate. Bars are means ± standard deviations (*n* = 5). Different lower-case letters (a–c) indicate statistically significant differences (*p* < 0.05).

The resilience of shrimp to environmental stress was assessed through a salinity challenge, in which salinity was reduced from 20 ppt to 5 ppt over 24 hours. Shrimp fed the CH and the KM diets exhibited the highest survival rates (89.8% and 88.1%, respectively) following the salinity challenge, which were significantly higher than those of shrimp fed the TH diet (84.4%) (**Figure 4B**). Survival rates for shrimp fed the CH and KM diets were also higher, however, not significantly different than the shrimp fed the CTRL diet. No significant differences were observed in survival rates between CTRL and shrimp fed the TH diet (**Figures 4A, B**).

This corresponded with the observations during the initial feeding trial, where survival rates were highest in shrimp fed the CH and SLM diets, significantly exceeding those fed the TH diet (**Figure 4A**).

To further investigate the physiological mechanisms underlying the observed improvements in stress resilience, immune response and oxidative stress markers were analyzed.

#### 3.2.2 Post-challenge immune response

**Figure 5:**
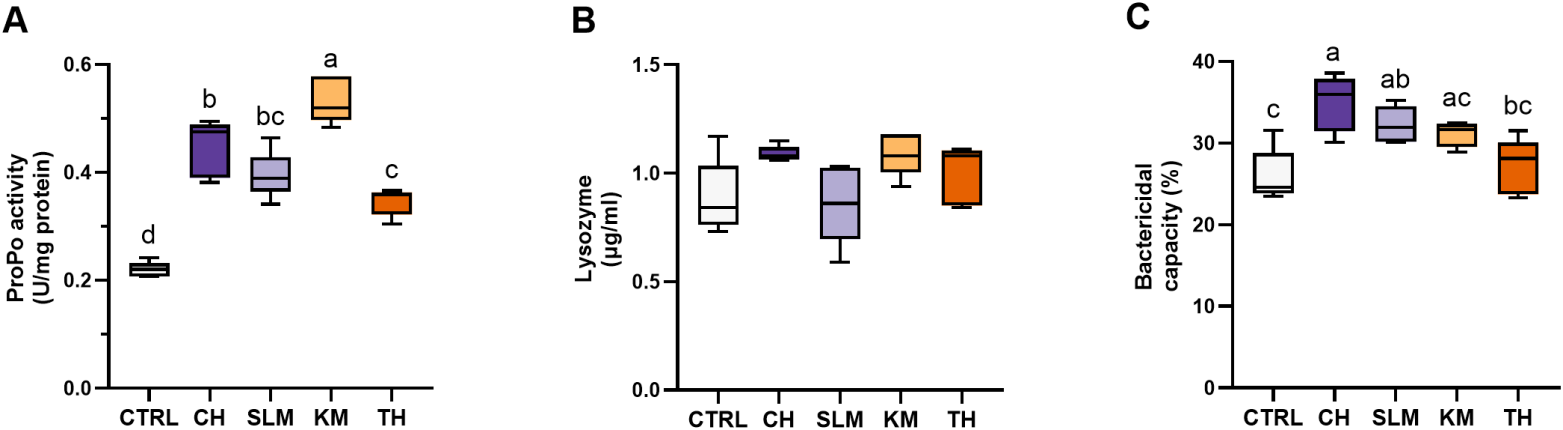
Hemolymph immune biomarkers following the salinity challenge (drop from 20 ppt to 5 ppt during 24h). **(A)** Prophenoloxidase activity. **(B)** Lysozyme. **(C)** Bactericidal capacity. CTRL, Control; CH, Calanus hydrolysate; SLM, Squid-liver meal; KM, Krill meal; TH, Tuna hydrolysate. Results presented with box (25th to 75th percentiles with line at median) and whiskers (min to max) plots (*n* = 5). Different lower-case letters (a–d) indicate statistically significant differences (*p* < 0.05).

Immune response markers were measured to assess the effects of the experimental diets on shrimp health. Shrimp fed the CH, KM, SLM and TH diets exhibited significantly higher ProPO compared to the CTRL diets, with the highest activity observed in shrimp fed KM followed by CH, SLM, and TH diets (**Figure 5A**). Bactericidal capacity was also highest in shrimp fed CH, significantly exceeding CTRL and TH (**Figure 5C**). No significant differences in lysozyme levels were observed among the dietary treatments (**Figure 5B**). The enhanced ProPO activity and antibacterial capacity observed in this study (especially evident for the shrimp fed the CH diet), suggest improved immune modulation, supporting resilience under hyposalinity stress.

#### 3.2.3 Oxidative stress biomarkers

**Figure 6:**
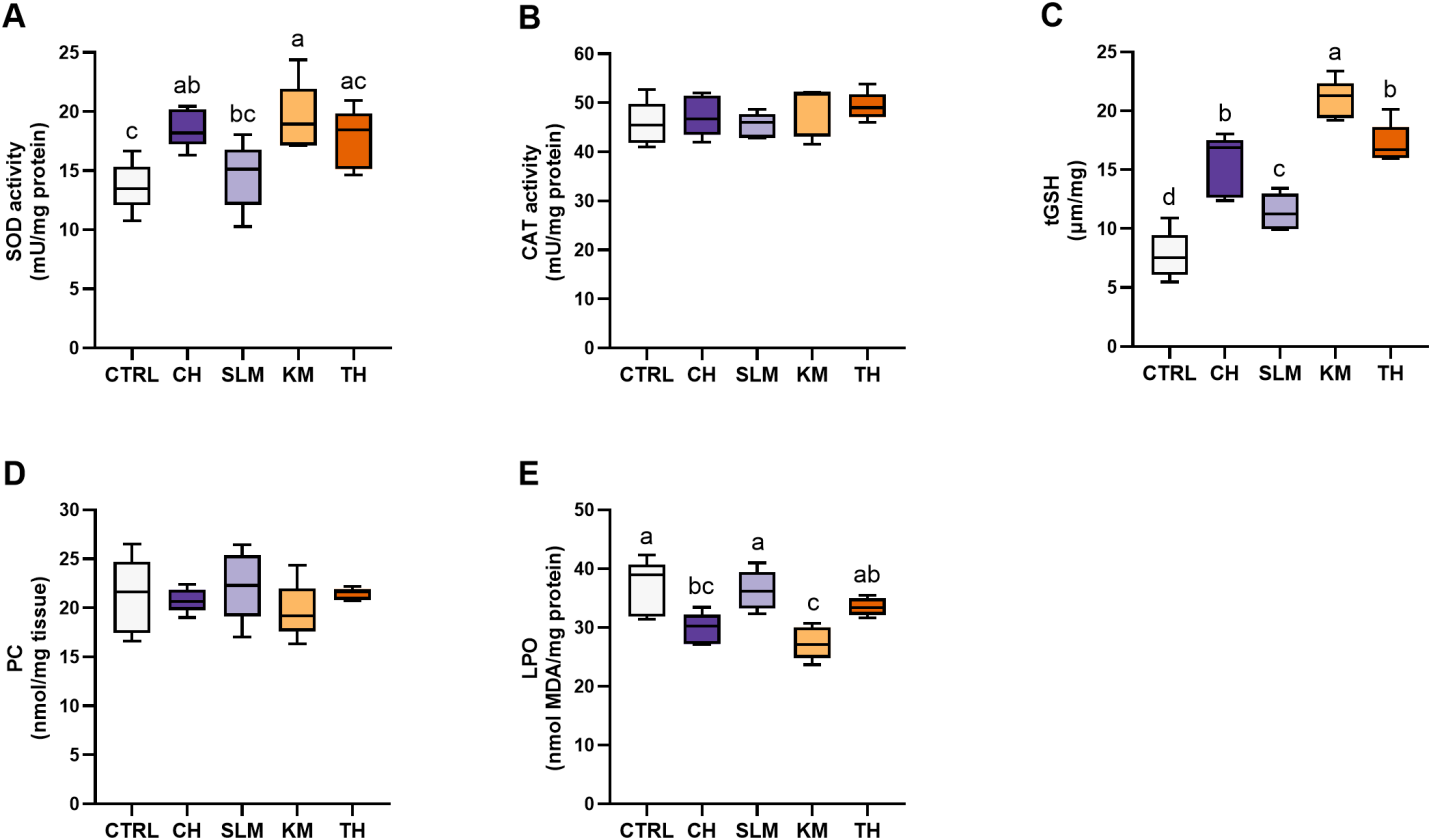
Oxidative stress biomarkers in whiteleg shrimp hepatopancreas collected seven days post-salinity challenge. **(A)** Superoxide dismutase activity. **(B)** Catalase activity. **(C)** Total glutathione. **(D)** Protein carbonyls. **(E)** Lipid peroxidation. CTRL, Control; CH, Calanus hydrolysate; SLM, Squid-liver meal; KM, Krill meal; TH, Tuna hydrolysate. Results presented with box (25th to 75th percentiles with line at median) and whiskers (min to max) plots (*n* = 5). Different lower-case letters (a–d) indicate statistically significant differences (*p* < 0.05).

Hepatopancreatic oxidative stress biomarkers were assessed seven days after the salinity challenge to determine the antioxidative effects of the experimental diets. Shrimp fed the CH diet and the KM diets showed significantly reduced lipid peroxidation levels (MDA) in the hepatopancreas compared to the CTRL and the SLM diets, as measured via TBARS assay (**Figure 6E**).

The antioxidant enzyme, SOD activity was significantly higher in shrimp fed the CH and KM diets compared to CTRL (**Figures 6A, C**). In addition, tGSH activity was significantly higher in shrimp fed all four test diets compared to the CTRL diet. No significant differences were observed in catalase (CAT) activity or protein carbonyl levels (**Figures 6B, D**). The moderate increases in SOD and tGSH antioxidant enzyme activities, evident for shrimp fed the CH and KM diets, demonstrate an adaptive response to oxidative stress.

#### 3.2.4 Amino acid metabolism, metabolic adaptation and stress

**Figure 7:**
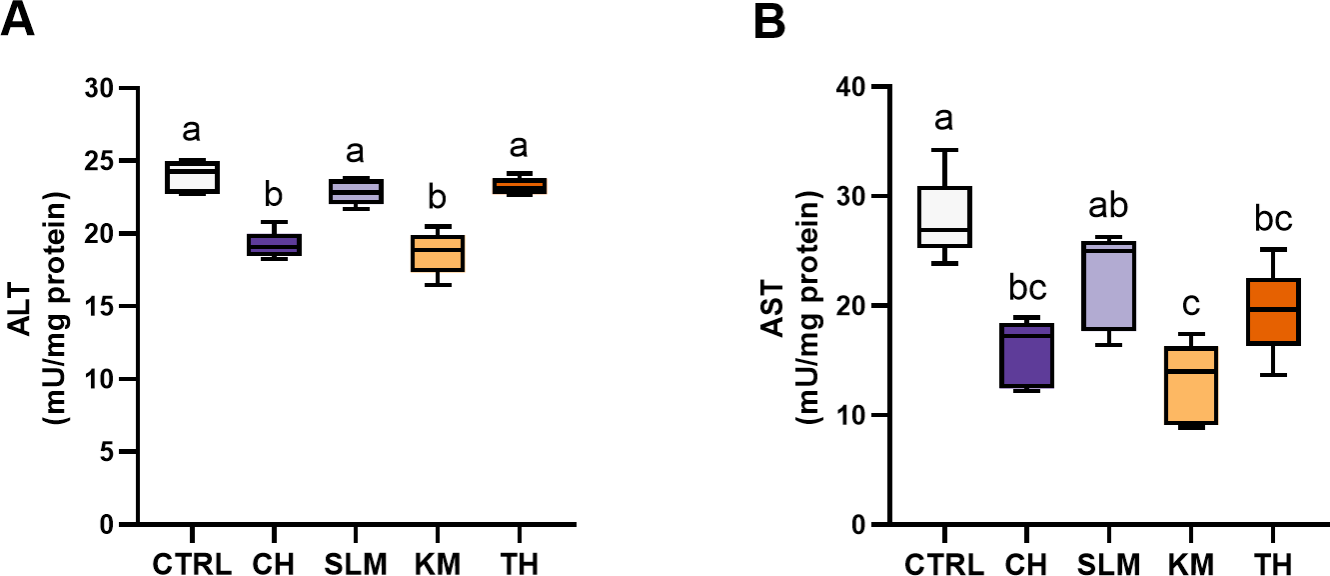
Hepatopancreatic enzyme activity in whiteleg shrimp post-salinity challenge. CTRL, Control; CH, Calanus hydrolysate; SLM, Squid-liver meal; KM, Krill meal; TH, Tuna hydrolysate. Results presented with box (25th to 75th percentiles with line at median) and whiskers (min to max) plots (*n* = 5). Different lower-case letters (a–c) indicate statistically significant differences (*p* < 0.05).

After the salinity challenge, hepatopancreatic ALT activity was significantly lower in shrimp fed CH and KM compared to shrimp fed all other diets (**Figure 7A**). AST activity was also lowest in shrimp fed KM, CH, and TH, all of which had significantly lower activity compared to the CTRL diet (**Figure 7B**). Downregulation of ALT and AST activity in shrimp fed CH and KM may indicate reduced gluconeogenesis, lower amino acid catabolism, metabolic stress, and less tissue damage.

After the hyposalinity challenge, free amino acid and nitrogen metabolite profiles in the hepatopancreas were significantly influenced by the diets (**Table 5**). Shrimp fed CH had the highest levels of total essential amino acids (EAA), while CTRL and TH diets resulted in significantly higher non-essential amino acid (NEAA) levels. Total nitrogen metabolites were highest in shrimp fed SLM and lowest in those fed KM.

**Table 5:** Free amino acids and nitrogen metabolites in shrimp hepatopancreas post-salinity challenge.

mg/g DM	CTRL	CH	SLM	KM	TH
Essential amino acids					
Arg	22.1 ± 1.3 ^a^	23.8 ± 1.4 ^a^	12.9 ± 1.4 ^c^	18.7 ± 1.4 ^b^	13.1 ± 1.0 ^c^
His	7.1 ± 0.4 ^b^	7.1 ± 0.8 ^b^	10.4 ± 1.5 ^a^	6.8 ± 1.0 ^b^	8.0 ± 0.8 ^b^
Ile	4.5 ± 0.6 ^b^	6.1 ± 1.1 ^a^	5.3 ± 0.4 ^ab^	5.2 ± 0.4 ^ab^	5.0 ± 0.5 ^ab^
Leu	12.7 ± 1.3 ^b^	15.9 ± 0.7 ^a^	16.1 ± 1.0 ^a^	15.5 ± 0.2 ^a^	15.2 ± 0.8 ^a^
Lys	13.8 ± 0.4 ^ab^	13.1 ± 0.5 ^b^	14.3 ± 0.5 ^a^	13.8 ± 0.2 ^ab^	13.6 ± 0.5 ^ab^
Thr	12.7 ± 1.3 ^a^	7.4 ± 0.5 ^b^	6.7 ± 0.7 ^bc^	5.0 ± 1.1 ^c^	5.1 ± 0.9 ^c^
Trp	2.8 ± 0.5 ^b^	3.5 ± 0.3 ^a^	3.1 ± 0.4 ^ab^	3.1 ± 0.3 ^ab^	3.7 ± 0.4 ^a^
Val	2.9 ± 0.4 ^b^	5.3 ± 0.5 ^a^	5.1 ± 0.7 ^a^	3.7 ± 0.6 ^b^	3.8 ± 0.6 ^b^
Met	7.2 ± 0.8	7.8 ± 0.4	6.8 ± 0.6	6.6 ± 1.3	8.2 ± 1.1
Cys	0.12 ± 0.02 ^b^	0.17 ± 0.02 ^a^	0.13 ± 0.02 ^b^	0.19 ± 0.02 ^a^	0.10 ± 0.02 ^b^
Phe	11.6 ± 0.3 ^b^	14.9 ± 0.9 ^a^	13.8 ± 1.6 ^a^	13.3 ± 0.8 ^ab^	13.8 ± 0.9 ^a^
Tyr	5.4 ± 0.1 ^c^	6.3 ± 0.4 ^b^	6.2 ± 0.1 ^b^	6.4 ± 0.1 ^b^	10.1 ± 0.2 ^a^
Total	102.92 ± 4.42 ^b^	111.49 ± 3.57 ^a^	100.85 ± 3.70 ^b^	98.22 ± 2.32 ^b^	99.71 ± 1.43 ^b^
Non-essential amino acids					
Asp	4.7 ± 0.2	5.0 ± 0.2	4.2 ± 0.5	4.8 ± 0.7	4.9 ± 0.9
Glu	20.4 ± 1.0 ^a^	12.7 ± 1.2 ^b^	9.9 ± 1.0 ^c^	12.3 ± 1.2 ^b^	19.4 ± 1.2 ^a^
Ala	14.6 ± 0.4 ^a^	8.3 ± 0.8 ^b^	9.2 ± 0.5 ^b^	8.6 ± 1.2 ^b^	14.4 ± 1.3 ^a^
Gly	11.8 ± 1.5 ^b^	8.9 ± 0.5 ^c^	11.1 ± 1.5 ^b^	7.3 ± 1.2 ^c^	15.4 ± 0.6 ^a^
Pro	12.0 ± 1.1 ^a^	8.7 ± 0.6 ^b^	12.1 ± 1.5 ^a^	7.9 ± 0.7 ^b^	12.1 ± 1.1 ^a^
Ser	16.5 ± 0.4 ^b^	16.2 ± 1.1 ^b^	17.9 ± 0.6 ^a^	16.7 ± 0.8 ^ab^	14.4 ± 0.3 ^c^
Total	79.83 ± 2.49 ^a^	59.75 ± 2.01 ^bc^	64.37 ± 3.51 ^b^	57.57 ± 2.40 ^c^	80.53 ± 1.79 ^a^
Nitrogen metabolites					
Asn	2.5 ± 0.1 ^b^	2.2 ± 0.3 ^b^	4.1 ± 0.1 ^a^	2.2 ± 0.1 ^b^	2.4 ± 0.1 ^b^
Gln	12.8 ± 0.8	11.7 ± 0.9	10.7 ± 1.4	10.6 ± 2.0	12.4 ± 0.8
Tau	5.6 ± 0.5 ^b^	3.6 ± 0.3 ^d^	8.3 ± 1.3 ^a^	4.1 ± 0.7 ^cd^	5.4 ± 0.7 ^bc^
Orn	2.8 ± 0.1 ^c^	3.6 ± 0.2 ^a^	3.3 ± 0.0 ^b^	2.9 ± 0.1 ^c^	2.9 ± 0.1 ^c^
GABA	0.41 ± 0.04 ^b^	0.70 ± 0.05 ^a^	0.70 ± 0.05 ^a^	0.74 ± 0.05 ^a^	0.69 ± 0.02 ^a^
HPro	0.19 ± 0.02 ^b^	0.40 ± 0.06 ^a^	0.37 ± 0.02 ^a^	0.12 ± 0.02 ^c^	0.22 ± 0.03 ^b^
BAla	0.22 ± 0.01 ^bc^	0.25 ± 0.02 ^ab^	0.28 ± 0.01 ^a^	0.21 ± 0.03 ^c^	0.24 ± 0.02 ^bc^
HCys	0.025 ± 0.001	0.027 ± 0.001	0.026 ± 0.000	0.026 ± 0.001	0.026 ± 0.001
Cysta	0.048 ± 0.002 ^c^	0.076 ± 0.001 ^a^	0.056 ± 0.001 ^b^	0.054 ± 0.006 ^b^	0.047 ± 0.001 ^c^
TMG	0.032 ± 0.001 ^b^	0.064 ± 0.003 ^a^	0.038 ± 0.004 ^b^	0.060 ± 0.002 ^a^	0.033 ± 0.001 ^bc^
SAM	1.10 ± 0.04 ^c^	1.39 ± 0.03 ^a^	1.07 ± 0.04 ^c^	1.24 ± 0.08 ^b^	1.10 ± 0.02 ^c^
SAH	0.18 ± 0.01	0.17 ± 0.03	0.16 ± 0.02	0.16 ± 0.01	0.18 ± 0.02
Total	25.92 ± 0.94 ^b^	24.14 ± 0.76 ^bc^	29.10 ± 1.43 ^a^	22.46 ± 2.27 ^c^	25.63 ± 0.97 ^b^

CTRL, Control; CH, Calanus hydrolysate; SLM, Squid-liver meal; KM, Krill meal; TH, Tuna hydrolysate. Values are means ± standard deviations (*n* = 5). Different lower-case letters (a–d) indicate statistically significant differences (*p* < 0.05).

Lower levels of the key osmolytes glycine, proline, glutamine, and taurine were evident in the hepatopancreas from the shrimp fed the CH and KM diets (**Table 5**). The depletion of these compounds is consistent with their active mobilization and release into the hemolymph to restore osmotic balance under hypo salinity stress, a well-documented mechanism in penaeid shrimp (Shinji et al., 2012; Chen et al., 2015).

## 4 Discussion

Shrimp aquaculture is under increasing pressure to address two critical challenges: reducing reliance on fishmeal and enhancing resilience to environmental stressors. This study evaluated the efficacy of CH as a functional feed ingredient for whiteleg shrimp (*L. vannamei*), focusing on its ability to compensate for a 50% reduction in fishmeal while improving growth performance, nutrient retention, and stress resilience. The findings demonstrate that CH enhances feed efficiency, antioxidant defenses, immune functionality, and metabolic adaptations, particularly under salinity stress. These results show that CH is a promising candidate for sustainable shrimp aquaculture and offer an introduction to the mechanistic foundation for its functional benefits.

### 4.1 Growth performance and feed efficiency at reduced fishmeal

Shrimp fed the CH-diet demonstrated improved feed conversion ratios (FCR) and protein efficiency ratios (PER), and robust growth performance, despite a 50% reduction in fishmeal compared to the CTRL shrimp. These findings directly address sustainability constraints in aquaculture, demonstrating that CH can maintain growth efficiency while reducing the dependency on fishmeal. Improved PER suggests improved protein utilization, while preserved specific growth rates (SGR) and desired final body weight align with the metabolic and immune benefits observed under salinity stress, as discussed later.

The strong performance of the shrimp fed the CH diet is consistent with previous studies on functional marine ingredients, such as krill meal and squid-liver meal, which have been shown to enhance feed efficiency and growth (Nunes et al., 2019; Wei et al., 2022). However, the lower performance of the TH diet highlights the variability in functional ingredient efficacy, which may be affected by differences in digestibility, palatability, or nutrient composition. Future research should conduct more detailed ingredient-specific assessments to identify the drivers of these differences and optimize feed formulations. Key areas of investigation should include the digestibility and bioavailability of amino acids across the test ingredients (CH, TH, KM, and SLM), particularly for indispensable amino acids where TH showed notably lower dietary concentrations. Also, the role of compounds that enhance palatability, such as free amino acids and nucleotides, should be investigated to possibly explain the differences in feed intake and performance.

### 4.2 Nutrient retention and whole-body composition

The shrimp fed the CH diet exhibited higher energy and lipid retention, along with improved whole-body protein retention, compared with the control diet. These general improvements clearly indicate that there are nutrient-dependent benefits associated with the intake of CH under reduced fishmeal conditions (**Figure 3**; **Table 4**). Elevated lipid retention may provide energy buffering during stress, while higher protein retention supports structural growth and metabolic capacity (Hossain et al., 2024; Li et al., 2025). Notably, the CTRL diet contained approximately double the fishmeal level of the CH, SLM, and KM diets, making the comparable or superior performance observed in these groups particularly relevant from a sustainability standpoint. These findings align with the discussed improvements in feed efficiency along with enhanced stress resilience, highlighting the integrated benefits of CH as a functional feed ingredient.

Even though squid-liver meal also showed elevated protein retention and energy, the performance of CH and krill meal was particularly notable given their ability to sustain growth and resilience at reduced fishmeal levels. However, retention calculations in this study relied on pooled samples and rationing adjustments, which may not capture individual-level variability. Hence, future studies should incorporate individual-level intake tracking and stable isotope labeling to refine nutrient flux and retention pathways.

### 4.3 Immune functionality and post-challenge survival

Shrimp fed the CH diet exhibited enhanced immune functionality, as evidenced by elevated activity and antibacterial capacity in the hemolymph. These immune enhancements were accompanied by the highest survival rates following the salinity challenge, significantly outperforming the control and TH diets. Prophenoloxidase is important in the melanization process involved in defense against pathogens and stress, and elevated ProPO activity indicates that the melanization defense mechanisms are strengthened. The increased antibacterial activity against Vibrio confirms that CH and KM strengthen the immune response and contributes to improved bacteriolysis and pathogen resistance.

These findings align well with previous research on functional marine ingredients, which have been shown to stimulate innate immune responses and improve disease resistance in shrimp (Costas et al., 2011). The combination of enhanced immune responses and improved survival rates indicates that CH (similar to KM) inclusion has the potential to enhance resilience to environmental stress, a critical factor for operational stability in aquaculture systems. Notably, shrimp fed the CH diet demonstrated superior immune responses compared to those fed TH, suggesting that the specific bioactive compounds in CH may offer distinct immunomodulatory benefits. However, the immune readouts of the current study are limited to hemolymph assays, which do not capture pathogen-specific responses or cellular immunity. Future research should therefore incorporate pathogen challenge models and cellular immunity markers to provide a more comprehensive understanding of immunomodulatory effects associated with inclusion of CH in the diet.

### 4.4 Antioxidant capacity and post-challenge oxidative injury

The inclusion of CH significantly enhanced the antioxidative defense, as evidenced by elevated SOD activity and tGSH levels, together with reduced LPO, in the hepatopancreas following salinity stress (**Figures 6A, C, E**). These findings indicate that CH inclusion helps mitigate oxidative damage, a critical factor in maintaining cellular integrity under environmental stress (Liu et al., 2007). The increased SOD activity may reflect a coordinated response to both oxidative and immune stimulation, consistent with the elevated ProPo activity observed in the same group. The elevated tGSH suggests strengthened glutathione-dependent detoxification pathways, while the reduced LPO confirms that lipid peroxidation was kept within subclinical thresholds despite the hypo salinity challenge (Ye et al., 2024). Furthermore, reduced ALT activity in shrimp fed the CH diet (**Figure 7A**) indicates lower hepatopancreas injury, further supporting the protective effects of CH during acute salinity stress. The low MDA levels observed in the hepatopancreas of CH- and KM-fed shrimp indicate that, despite salinity-induced oxidative pressure, lipid peroxidation was kept within subclinical thresholds.

These results are also consistent with prior studies demonstrating the antioxidative and immunostimulatory benefits of krill meal in compound aquaculture diets (Nunes et al., 2019; Wei et al., 2022; Tor.wattanaphol et al., 2026), further supporting the value of low-trophic marine ingredients as functional alternatives to fishmeal. CH and KM inclusion resulted in outcomes that were more or less identical in our trial. However, the evidence from our study demonstrates stress mitigation at reduced fishmeal levels for the shrimp fed the CH and KM diets. This suggests that CH not only compensates for the loss of fishmeal but also enhances the physiological capacity to withstand oxidative stress after inclusion of CH and KM. These results are promising, yet the absence of histological evaluations limits the ability to confirm tissue-level protection. Future studies should also incorporate histopathological analyses, supported by enzyme activity kinetic studies, to further elucidate the mechanisms underlying the antioxidative effects associated with CH and KM inclusion.

### 4.5 Metabolic adaptations under salinity stress

The metabolic profiles of shrimp fed the CH and KM diets were very similar, indicating significant adaptations to salinity stress (**Table 5**). Elevated levels of GABA, S-adenosylmethionine (SAM), and sulfur-containing amino acids (cysteine and methionine) suggest coordinated biochemical responses supporting antioxidative capacity and stress signaling (Bae et al., 2022; Chuphal et al., 2025). Alongside these elevations, reduced hepatopancreatic levels of taurine, alanine, glycine, and proline (**Table 5**) are consistent with their mobilization as osmolytes during acute salinity stress (Shinji et al., 2012).

These metabolic signatures correspond to the physiological demands of salinity adaptation, in which sulfur amino acids contribute to glutathione synthesis and redox buffering, while GABA and SAM support nitrogen metabolism, methylation reactions and stress signaling (Bae et al., 2022; Chuphal et al., 2025). The observed retention of cysteine and methionine further supports the potential of the CH and KM diets to stimulate the sulfur-based antioxidative pathways. These findings suggest that the inclusion of CH and KM both support metabolic changes that enable the shrimp to maintain homeostasis under stress. However, we did not conduct hemolymph osmolarity and ion transport measurements, our study cannot directly link the changes in taurine and amino acid metabolism to osmoregulation. Future studies should preferably integrate studies on osmoregulatory assays and targeted metabolomics to resolve the biochemical pathways underlying stress-adaptive effects associated with inclusion of CH in the diet.

### 4.6 Sustainability implications

The ability of inclusion of CH to compensate for the 50% reduction in fishmeal while maintaining growth performance and strengthening resilience emphasizes its potential as a sustainable feed ingredient. *C. finmarchicus* represents a highly abundant and underutilized marine resource, with an annual biomass exceeding 290 million tonnes in the Norwegian Sea (Skjoldal, 2004). Harnessing this low-trophic-level organism is supported by ecosystem-based resource management and balanced harvesting (Zhou et al., 2019).

The production of CH is supported by advances in harvesting and processing technologies, as well as precautionary management frameworks that ensure sustainable use of *C. finmarchicus* populations (Albrektsen et al., 2022; Fjeld et al., 2023). However, further investigations, including environmental and economic assessments of the harvest of *C. finmarchicus* and CH production, are needed to ensure an ecologically and commercially viable development of *C. finmarchicus* and CH as feed ingredients.

### 4.7 Limitations and future directions

This study provides compelling evidence for the efficacy of CH as a functional feed ingredient, but several limitations must be acknowledged. First, this study only includes one selected concentration of the hydrolysates and does not indicate whether this is the optimal concentration to be used. The 62-day trial may not capture long-term physiological and production outcomes under reduced fishmeal conditions, as the full production/growth cycle is somewhat longer. Additionally, the absence of histological evaluations and osmoregulatory measurements limits the mechanistic interpretation of tissue protection and amino acid mobilization under salinity stress. Finally, the single facility and shrimp genetic line used in this study may limit the generalizability of these observations. It should also be noted that hemolymph and hepatopancreas samples were collected from only 2 shrimp per replicate tank (n = 10 per dietary treatment), which was constrained by the terminal nature of hemolymph collection and the use of the same tanks for both the growth trial and salinity challenge. While this sample size is consistent with comparable published shrimp challenge studies, the inherent individual variability of parameters such as ProPo activity may limit the statistical sensitivity of the immune and oxidative stress analyses. Future challenge trials should aim for higher per-tank sampling to improve statistical power for these endpoints.

Future research should address these limitations by incorporating various CH inclusion concentrations, histological assessments, osmoregulatory endpoints, and targeted metabolomics to delineate stress-adaptation pathways. Long-term and multi-site trials are also needed to validate and confirm the efficacy of CH inclusion across production cycles, facilities, and different genetic shrimp lines. Additionally, dose-response studies and formulation optimization will be critical for maximizing the functional benefits of CH in commercial shrimp farming, ultimately supporting more sustainable aquaculture practices. Beyond its evaluation as a standalone fishmeal replacer, the present findings suggest that CH has potential as a functional co-ingredient in combination with other alternative protein sources, such as insect meal, single-cell protein, or microbial fermentation products. In this role, CH could contribute bioactive compounds including free amino acids and peptides that complement the amino acid profiles of plant-based or terrestrial protein sources, while collectively reducing reliance on soybean meal.

## 5 Conclusions

This study indicates that *C. finmarchicus* hydrolysate and krill meal can compensate effectively for a 50% reduction in fishmeal while enhancing antioxidant defenses, immune function, and metabolic adaptations under salinity stress. These mechanistic improvements correspond with improved feed efficiency, nutrient retention, and resilience, supporting *C. finmarchicus* and CH as sustainable and functional feed ingredients for shrimp aquaculture.

## Data Availability

The original contributions presented in the study are included in the article/**Supplementary Material**. Further inquiries can be directed to the corresponding author.
