# Supplementary material for: The functional effects of Calanus finmarchicus hydrolysate as a novel feed ingredient for whiteleg shrimp (Litopenaeus vannamei)
Source: Front Physiol. 2026 Jun 1;17:1820807. doi: 10.3389/fphys.2026.1820807 (PMC13265333; doi:10.3389/fphys.2026.1820807)
Supplement: Supplementary file 1 [file Table1.docx]

Supplementary Material

**Supplementary Table S1.** Compositional analyses of the feeds.

**Supplementary Table S2.** Water parameters during initial trial and salinity challenge.

**Supplementary Table S3.** Zootechnical data initial trial (62 days).

**Supplementary Table S4.** Whole-body composition of the shrimp after 62 days.

**Supplementary Table S5.** Nutrient retention of the shrimp after 62 days.

**Supplementary Table S6.** Survival after salinity test.

**Supplementary Table S7.** Immune criteria after the salinity test.

**Supplementary Table S8.** Oxidative stress parameters after the salinity test.

**Supplementary Table S9.** Metabolic status after the salinity test.

**Supplementary Table S10.** Statistical analyses (ANOVA, Brown-Forsythe, Tukey post-hoc).

**Supplementary Table S1.** Compositional analyses of the feeds.

| **As fed basis** | **%** | **%** | | **%** | **%** | **kJ/g** | | **%** |  |  |  |  |
| --- | --- | --- | --- | --- | --- | --- | --- | --- | --- | --- | --- | --- |
|  | **Moisture** | **Ash** | | **Protein** | **Lipid** | **Energy** | | **P** |  |  |  |  |
| CTRL | 6.67 | 9.12 | | 32.64 | 7.00 | 18.34 | | 1.04 |  |  |  |  |
| CTRL | 6.64 | 9.18 | | 32.68 | 7.23 | 18.38 | | 1.02 |  |  |  |  |
| CALANUS | 6.55 | 8.04 | | 32.62 | 7.02 | 18.43 | | 0.95 |  |  |  |  |
| CALANUS | 6.52 | 8.02 | | 32.83 | 7.22 | 18.30 | | 0.93 |  |  |  |  |
| SQUID | 6.39 | 8.15 | | 32.78 | 7.02 | 18.41 | | 0.99 |  |  |  |  |
| SQUID | 6.42 | 8.24 | | 32.70 | 7.13 | 18.30 | | 1.03 |  |  |  |  |
| KRILL | 6.17 | 8.64 | | 32.67 | 7.10 | 18.37 | | 1.05 |  |  |  |  |
| KRILL | 6.21 | 8.64 | | 32.79 | 7.20 | 18.50 | | 0.99 |  |  |  |  |
| TUNA | 6.64 | 8.51 | | 32.74 | 7.15 | 18.50 | | 0.92 |  |  |  |  |
| TUNA | 6.60 | 8.45 | | 32.67 | 7.22 | 18.46 | | 0.94 |  |  |  |  |
| **As fed basis** | **%** | **%** | **%** | **%** | **%** | **%** | **%** | **%** | **%** | **%** | **%** | **%** |
|  | **Arg** | **His** | **Ile** | **Leu** | **Lys** | **Thr** | **Trp** | **Val** | **Met** | **Cys** | **Phe** | **Tyr** |
| CTRL | 2.10 | 0.79 | 1.28 | 2.36 | 2.18 | 1.27 | 0.41 | 1.58 | 0.74 | 0.47 | 1.46 | 1.06 |
| CTRL | 2.07 | 0.77 | 1.27 | 2.31 | 2.21 | 1.31 | 0.42 | 1.52 | 0.71 | 0.49 | 1.43 | 1.04 |
| CALANUS | 2.00 | 0.74 | 1.30 | 2.33 | 2.13 | 1.21 | 0.41 | 1.50 | 0.71 | 0.50 | 1.47 | 1.01 |
| CALANUS | 2.03 | 0.77 | 1.28 | 2.34 | 2.18 | 1.24 | 0.43 | 1.53 | 0.72 | 0.51 | 1.49 | 1.06 |
| SQUID | 2.08 | 0.80 | 1.30 | 2.36 | 2.16 | 1.27 | 0.41 | 1.52 | 0.76 | 0.50 | 1.52 | 1.14 |
| SQUID | 2.05 | 0.82 | 1.27 | 2.31 | 2.12 | 1.24 | 0.41 | 1.53 | 0.74 | 0.51 | 1.50 | 1.11 |
| KRILL | 2.10 | 0.79 | 1.31 | 2.38 | 2.20 | 1.27 | 0.40 | 1.51 | 0.73 | 0.48 | 1.56 | 1.06 |
| KRILL | 2.08 | 0.77 | 1.35 | 2.35 | 2.17 | 1.29 | 0.41 | 1.48 | 0.72 | 0.49 | 1.57 | 1.04 |
| TUNA | 2.01 | 0.74 | 1.25 | 2.26 | 2.10 | 1.22 | 0.39 | 1.45 | 0.67 | 0.46 | 1.42 | 1.06 |
| TUNA | 2.02 | 0.73 | 1.21 | 2.28 | 2.13 | 1.25 | 0.40 | 1.46 | 0.69 | 0.49 | 1.44 | 1.07 |
| **As fed basis** | **%** | **%** | **%** | **%** | **%** | **%** |  |  |  |  |  |  |
|  | **Asp** | **Glu** | **Ala** | **Gly** | **Pro** | **Ser** |  |  |  |  |  |  |
| CTRL | 2.89 | 6.23 | 1.70 | 2.03 | 2.05 | 1.57 |  |  |  |  |  |  |
| CTRL | 2.97 | 6.15 | 1.65 | 2.04 | 2.02 | 1.62 |  |  |  |  |  |  |
| CALANUS | 2.89 | 6.27 | 1.66 | 2.15 | 2.16 | 1.57 |  |  |  |  |  |  |
| CALANUS | 2.93 | 6.32 | 1.71 | 2.18 | 2.14 | 1.60 |  |  |  |  |  |  |
| SQUID | 3.11 | 6.17 | 1.63 | 1.85 | 1.92 | 1.57 |  |  |  |  |  |  |
| SQUID | 3.06 | 6.20 | 1.59 | 1.89 | 1.96 | 1.61 |  |  |  |  |  |  |
| KRILL | 3.03 | 6.29 | 1.61 | 1.96 | 2.11 | 1.59 |  |  |  |  |  |  |
| KRILL | 3.10 | 6.34 | 1.64 | 1.95 | 2.05 | 1.60 |  |  |  |  |  |  |
| TUNA | 2.94 | 6.12 | 1.57 | 1.87 | 2.06 | 1.53 |  |  |  |  |  |  |
| TUNA | 2.97 | 6.08 | 1.60 | 1.84 | 2.08 | 1.57 |  |  |  |  |  |  |

**Supplementary Table S2.** Water parameters during initial trial and salinity challenge.

| **Date** | **Days** | **Temperature (ºC)** | **Dissolved oxygen (mg/L)** | **Salinity (‰)** | **pH** | **NH3 (mg/L)** |  |
| --- | --- | --- | --- | --- | --- | --- | --- |
| 15-07-2021 | 0 | 28.0 | 7.5 | 20.5 | 8.0 | 0.003 | **Growth performance** |
| 16-07-2021 | 1 | 28.7 | 7.2 | 20.2 | 7.8 |  |  |
| 17-07-2021 | 2 | 28.7 | 6.7 | 20.5 | 8.0 |  |  |
| 18-07-2021 | 3 | 28.8 | 6.5 | 20.6 | 8.0 |  |  |
| 19-07-2021 | 4 | 28.6 | 7.0 | 20.4 | 7.8 | 0.004 |  |
| 20-07-2021 | 5 | 28.7 | 6.7 | 20.7 | 7.8 |  |  |
| 21-07-2021 | 6 | 28.4 | 6.6 | 20.6 | 7.8 |  |  |
| 22-07-2021 | 7 | 28.3 | 6.6 | 20.5 | 7.8 |  |  |
| 23-07-2021 | 8 | 27.8 | 6.9 | 20.5 | 7.8 | 0.003 |  |
| 24-07-2021 | 9 | 28.0 | 6.7 | 20.4 | 7.9 |  |  |
| 25-07-2021 | 10 | 28.0 | 6.8 | 20.3 | 7.9 |  |  |
| 26-07-2021 | 11 | 28.3 | 6.7 | 20.3 | 7.8 |  |  |
| 27-07-2021 | 12 | 28.6 | 7.1 | 20.3 | 7.8 |  |  |
| 28-07-2021 | 13 | 28.5 | 6.7 | 20.1 | 7.8 | 0.002 |  |
| 29-07-2021 | 14 | 28.5 | 7.0 | 20.2 | 7.8 |  |  |
| 30-07-2021 | 15 | 28.4 | 6.5 | 20.2 | 7.8 |  |  |
| 31-07-2021 | 16 | 28.2 | 6.9 | 20.2 | 7.8 |  |  |
| 01-08-2021 | 17 | 28.0 | 7.1 | 20.1 | 7.8 | 0.004 |  |
| 02-08-2021 | 18 | 27.7 | 7.2 | 20.7 | 7.7 |  |  |
| 03-08-2021 | 19 | 28.0 | 6.7 | 20.4 | 7.8 |  |  |
| 04-08-2021 | 20 | 28.4 | 6.7 | 20.1 | 7.8 |  |  |
| 05-08-2021 | 21 | 28.6 | 6.5 | 20.0 | 7.8 |  |  |
| 06-08-2021 | 22 | 28.7 | 6.6 | 20.1 | 7.9 | 0.004 |  |
| 07-08-2021 | 23 | 28.6 | 6.2 | 20.0 | 7.9 |  |  |
| 08-08-2021 | 24 | 28.3 | 6.1 | 19.9 | 7.8 |  |  |
| 09-08-2021 | 25 | 28.1 | 6.5 | 19.9 | 7.8 |  |  |
| 10-08-2021 | 26 | 28.3 | 6.5 | 19.9 | 7.8 |  |  |
| 11-08-2021 | 27 | 28.5 | 6.3 | 19.8 | 7.9 |  |  |
| 12-08-2021 | 28 | 28.6 | 6.5 | 19.9 | 7.9 |  |  |
| 13-08-2021 | 29 | 28.2 | 6.5 | 19.7 | 8.0 | 0.002 |  |
| 14-08-2021 | 30 | 28.0 | 6.4 | 19.7 | 7.9 |  |  |
| 15-08-2021 | 31 | 27.9 | 6.3 | 19.7 | 7.9 |  |  |
| 16-08-2021 | 32 | 28.1 | 5.9 | 19.7 | 7.9 |  |  |
| 17-08-2021 | 33 | 27.9 | 5.9 | 19.7 | 7.9 |  |  |
| 18-08-2021 | 34 | 27.9 | 6.1 | 19.7 | 7.9 | 0.004 |  |
| 19-08-2021 | 35 | 27.7 | 5.9 | 19.7 | 7.9 |  |  |
| 20-08-2021 | 36 | 27.8 | 6.2 | 19.7 | 7.9 |  |  |
| 21-08-2021 | 37 | 28.3 | 6.2 | 19.7 | 7.7 |  |  |
| 22-08-2021 | 38 | 28.5 | 6.2 | 19.7 | 7.8 |  |  |
| 23-08-2021 | 39 | 28.5 | 6.1 | 19.7 | 7.8 | 0.005 |  |
| 24-08-2021 | 40 | 28.6 | 6.1 | 19.6 | 7.8 |  |  |
| 25-08-2021 | 41 | 28.5 | 6.0 | 19.6 | 7.6 |  |  |
| 26-08-2021 | 42 | 28.5 | 6.3 | 19.5 | 7.8 |  |  |
| 27-08-2021 | 43 | 28.3 | 6.1 | 19.4 | 7.7 |  |  |
| 28-08-2021 | 44 | 28.5 | 6.4 | 19.4 | 7.8 | 0.002 |  |
| 29-08-2021 | 45 | 28.7 | 6.6 | 19.4 | 7.8 |  |  |
| 30-08-2021 | 46 | 28.6 | 6.1 | 19.4 | 7.9 |  |  |
| 31-08-2021 | 47 | 28.4 | 6.4 | 19.3 | 7.9 |  |  |
| 01-09-2021 | 48 | 28.7 | 6.1 | 19.3 | 7.9 |  |  |
| 02-09-2021 | 49 | 28.8 | 6.4 | 19.3 | 7.9 | 0.000 |  |
| 03-09-2021 | 50 | 28.6 | 6.7 | 19.7 | 8.1 |  |  |
| 04-09-2021 | 51 | 28.2 | 6.5 | 20.2 | 8.0 |  |  |
| 05-09-2021 | 52 | 28.6 | 7.0 | 20.2 | 7.9 |  |  |
| 06-09-2021 | 53 | 28.8 | 6.8 | 19.8 | 7.9 |  |  |
| 07-09-2021 | 54 | 28.6 | 6.9 | 20.2 | 7.9 | 0.003 |  |
| 08-09-2021 | 55 | 28.8 | 5.9 | 20.3 | 7.9 |  |  |
| 09-09-2021 | 56 | 28.6 | 7.4 | 20.4 | 7.8 |  |  |
| 10-09-2021 | 57 | 28.6 | 7.9 | 20.4 | 8.0 |  |  |
| 11-09-2021 | 58 | 28.3 | 7.1 | 20.2 | 7.7 |  |  |
| 12-09-2021 | 59 | 28.2 | 7.2 | 20.4 | 7.7 | 0.000 |  |
| 13-09-2021 | 60 | 28.5 | 7.0 | 20.4 | 7.7 |  |  |
| 14-09-2021 | 61 | 28.2 | 7.2 | 20.2 | 7.6 |  |  |
| 15-09-2021 | 62 | 27.9 | 7.3 | 20.3 | 7.7 | 0.002 |  |
| 16-09-2021 | 63 | 27.0 | 7.6 | 6.7 | 7.7 |  | **Salinity challenge** |
| 17-09-2021 | 64 | 27.1 | 7.5 | 5.8 | 7.6 |  |  |
| 18-09-2021 | 65 | 26.9 | 7.4 | 5.6 | 7.5 |  |  |
| 19-09-2021 | 66 | 27.4 | 7.6 | 5.4 | 7.5 |  |  |
| 20-09-2021 | 67 | 27.1 | 7.9 | 5.4 | 7.5 |  |  |
| 21-09-2021 | 68 | 27.3 | 7.8 | 5.4 | 7.6 | 0.003 |  |
| 22-09-2021 | 69 | 27.3 | 7.7 | 5.4 | 7.6 |  |  |
| **Average** |  | ***28.4*** | ***6.6*** | ***20.0*** | ***7.8*** |  |  |
| **STDEV** |  | ***0.3*** | ***0.4*** | ***0.4*** | ***0.1*** |  |  |
| **Max** |  | ***28.8*** | ***7.9*** | ***20.7*** | ***8.1*** |  |  |
| **Min** |  | ***27.7*** | ***5.9*** | ***19.3*** | ***7.6*** |  |  |

**Supplementary Table S3.** Zootechnical data initial trial (62 days).

|  |  |  | **g** |  | **g** |  | **%** | **g** | **g** | **g** | **g** | **%/day** | **g** |  | **%ABW/day** |  |
| --- | --- | --- | --- | --- | --- | --- | --- | --- | --- | --- | --- | --- | --- | --- | --- | --- |
|  | **Tank** | **Initial** | **Initial** | **Dead** | **Dead** | **Final** | **Survival** | **Final** | **IBW** | **FBW** | **Weight** | **SGR** | **Feed** | **FCR** | **Feed** | **PER** |
|  |  | **Shrimp** | **Biomass** |  | **Weight** | **Shrimp** |  | **Biomass** |  |  | **Gain** |  |  |  | **Intake** |  |
| CTRL | 7 | 80 | 171.6 | 7 | 60.9 | 73 | 91.3 | 1071.8 | 2.15 | 14.68 | 961.1 | 3.10 | 2057 | 2.14 | 5.34 | 1.43 |
| CTRL | 11 | 80 | 171.9 | 11 | 79.5 | 69 | 86.3 | 945.4 | 2.15 | 13.70 | 853.0 | 2.99 | 1816 | 2.13 | 5.24 | 1.44 |
| CTRL | 15 | 80 | 171.0 | 12 | 71.6 | 68 | 85.0 | 947.4 | 2.14 | 13.93 | 848.0 | 3.02 | 1793 | 2.11 | 5.17 | 1.45 |
| CTRL | 17 | 80 | 171.7 | 10 | 62.8 | 70 | 87.5 | 991.2 | 2.15 | 14.16 | 882.3 | 3.04 | 1818 | 2.06 | 5.04 | 1.49 |
| CTRL | 23 | 80 | 171.5 | 12 | 83.4 | 68 | 85.0 | 1006.4 | 2.14 | 14.80 | 918.3 | 3.12 | 1856 | 2.02 | 5.08 | 1.51 |
| CALANUS | 8 | 80 | 171.6 | 12 | 78.9 | 68 | 85.0 | 991.2 | 2.15 | 14.58 | 898.5 | 3.09 | 1673 | 1.86 | 4.64 | 1.64 |
| CALANUS | 9 | 80 | 171.9 | 8 | 57.7 | 72 | 90.0 | 1036.6 | 2.15 | 14.40 | 922.4 | 3.07 | 1767 | 1.92 | 4.72 | 1.60 |
| CALANUS | 13 | 80 | 171.8 | 7 | 42.6 | 73 | 91.3 | 1063.8 | 2.15 | 14.57 | 934.6 | 3.09 | 1632 | 1.75 | 4.26 | 1.75 |
| CALANUS | 20 | 80 | 171.5 | 9 | 68.6 | 71 | 88.8 | 1052.4 | 2.14 | 14.82 | 949.5 | 3.12 | 1781 | 1.88 | 4.69 | 1.63 |
| CALANUS | 24 | 80 | 171.4 | 10 | 74.6 | 70 | 87.5 | 1038.8 | 2.14 | 14.84 | 942.0 | 3.12 | 1722 | 1.83 | 4.59 | 1.67 |
| SQUID | 3 | 80 | 171.7 | 5 | 38.6 | 75 | 93.8 | 1073.8 | 2.15 | 14.32 | 940.7 | 3.06 | 1813 | 1.93 | 4.70 | 1.58 |
| SQUID | 5 | 80 | 171.9 | 8 | 67.8 | 72 | 90.0 | 1023.0 | 2.15 | 14.21 | 918.9 | 3.05 | 1716 | 1.87 | 4.63 | 1.64 |
| SQUID | 18 | 80 | 172.0 | 13 | 109.0 | 67 | 83.8 | 1013.4 | 2.15 | 15.13 | 950.4 | 3.15 | 1768 | 1.86 | 4.81 | 1.64 |
| SQUID | 19 | 80 | 171.6 | 11 | 84.3 | 69 | 86.3 | 1035.4 | 2.15 | 15.01 | 948.1 | 3.14 | 1806 | 1.90 | 4.83 | 1.60 |
| SQUID | 22 | 80 | 171.8 | 10 | 81.3 | 70 | 87.5 | 994.4 | 2.15 | 14.21 | 903.9 | 3.05 | 1737 | 1.92 | 4.80 | 1.59 |
| KRILL | 4 | 80 | 171.4 | 11 | 81.1 | 69 | 86.3 | 1006.8 | 2.14 | 14.59 | 916.5 | 3.09 | 1607 | 1.75 | 4.40 | 1.74 |
| KRILL | 12 | 80 | 171.3 | 14 | 94.1 | 66 | 82.5 | 934.6 | 2.14 | 14.16 | 857.4 | 3.05 | 1674 | 1.95 | 4.88 | 1.56 |
| KRILL | 14 | 80 | 172.3 | 12 | 85.0 | 68 | 85.0 | 948.8 | 2.15 | 13.95 | 861.5 | 3.01 | 1582 | 1.84 | 4.55 | 1.66 |
| KRILL | 21 | 80 | 172.1 | 9 | 78.9 | 71 | 88.8 | 1011.4 | 2.15 | 14.25 | 918.2 | 3.05 | 1767 | 1.92 | 4.82 | 1.59 |
| KRILL | 25 | 80 | 170.9 | 9 | 67.0 | 71 | 88.8 | 972.8 | 2.14 | 13.70 | 868.9 | 3.00 | 1653 | 1.90 | 4.66 | 1.61 |
| TUNA | 1 | 80 | 171.9 | 14 | 64.9 | 66 | 82.5 | 782.2 | 2.15 | 11.85 | 675.2 | 2.75 | 1507 | 2.23 | 5.10 | 1.37 |
| TUNA | 2 | 80 | 171.6 | 13 | 83.5 | 67 | 83.8 | 866.0 | 2.15 | 12.93 | 777.9 | 2.90 | 1678 | 2.16 | 5.22 | 1.42 |
| TUNA | 6 | 80 | 172.0 | 15 | 114.4 | 65 | 81.3 | 806.8 | 2.15 | 12.41 | 749.2 | 2.83 | 1554 | 2.07 | 5.12 | 1.47 |
| TUNA | 10 | 80 | 171.7 | 16 | 96.9 | 64 | 80.0 | 816.6 | 2.15 | 12.76 | 741.8 | 2.88 | 1652 | 2.23 | 5.39 | 1.37 |
| TUNA | 16 | 80 | 172.4 | 12 | 91.2 | 68 | 85.0 | 900.2 | 2.16 | 13.24 | 819.0 | 2.93 | 1673 | 2.04 | 5.03 | 1.50 |

**Supplementary Table S4.** Whole-body composition of the shrimp after 62 days.

| **Fresh basis** |  | **%** | **%** | **%** | **%** | **kJ/g** |
| --- | --- | --- | --- | --- | --- | --- |
|  | **Tank** | **Moisture** | **Ash** | **Protein** | **Fat** | **Energy** |
| INITIAL |  | 81.98 | 3.37 | 11.82 | 0.58 | 3.15 |
| INITIAL |  | 82.18 | 3.67 | 11.79 | 0.58 | 3.15 |
| CTRL | 7 | 76.20 | 2.88 | 17.36 | 0.90 | 4.60 |
| CTRL | 7 | 76.15 | 2.95 | 17.40 | 0.90 | 4.68 |
| CTRL | 11 | 75.99 | 2.91 | 17.39 | 0.97 | 4.78 |
| CTRL | 11 | 76.15 | 2.85 | 17.39 | 0.98 | 4.76 |
| CTRL | 15 | 76.34 | 2.64 | 17.36 | 0.97 | 4.57 |
| CTRL | 15 | 76.12 | 2.75 | 17.41 | 0.99 | 4.70 |
| CTRL | 17 | 76.51 | 3.05 | 17.10 | 0.91 | 4.67 |
| CTRL | 17 | 76.62 | 2.97 | 17.11 | 0.96 | 4.51 |
| CTRL | 23 | 76.20 | 2.78 | 17.41 | 1.12 | 4.72 |
| CTRL | 23 | 76.14 | 2.79 | 17.34 | 1.08 | 4.80 |
| CALANUS | 8 | 76.30 | 2.96 | 16.79 | 1.23 | 4.68 |
| CALANUS | 8 | 76.39 | 2.95 | 16.74 | 1.21 | 4.70 |
| CALANUS | 9 | 76.00 | 2.91 | 17.17 | 1.23 | 4.86 |
| CALANUS | 9 | 75.81 | 3.05 | 17.18 | 1.24 | 4.82 |
| CALANUS | 13 | 77.21 | 3.07 | 16.64 | 0.98 | 4.53 |
| CALANUS | 13 | 77.22 | 3.07 | 16.62 | 0.94 | 4.43 |
| CALANUS | 20 | 76.38 | 3.00 | 17.14 | 1.12 | 4.73 |
| CALANUS | 20 | 76.52 | 2.91 | 17.15 | 1.13 | 4.72 |
| CALANUS | 24 | 76.34 | 3.05 | 16.94 | 1.08 | 4.69 |
| CALANUS | 24 | 76.45 | 3.06 | 16.86 | 1.07 | 4.70 |
| SQUID | 3 | 76.53 | 2.98 | 16.78 | 0.85 | 4.59 |
| SQUID | 3 | 76.47 | 3.06 | 16.79 | 0.87 | 4.56 |
| SQUID | 5 | 76.88 | 3.12 | 16.93 | 0.94 | 4.52 |
| SQUID | 5 | 76.84 | 3.19 | 16.92 | 0.91 | 4.68 |
| SQUID | 18 | 76.90 | 3.02 | 16.80 | 0.90 | 4.51 |
| SQUID | 18 | 76.66 | 3.14 | 16.76 | 0.88 | 4.50 |
| SQUID | 19 | 75.25 | 3.09 | 18.06 | 1.06 | 4.94 |
| SQUID | 19 | 75.05 | 3.19 | 18.04 | 1.08 | 4.92 |
| SQUID | 22 | 76.35 | 3.05 | 17.09 | 1.04 | 4.55 |
| SQUID | 22 | 76.30 | 3.12 | 17.02 | 0.96 | 4.61 |
| KRILL | 4 | 75.50 | 2.97 | 17.69 | 1.25 | 4.88 |
| KRILL | 4 | 75.45 | 2.95 | 17.71 | 1.24 | 4.90 |
| KRILL | 12 | 75.48 | 3.22 | 17.98 | 1.26 | 4.94 |
| KRILL | 12 | 75.30 | 3.26 | 18.04 | 1.25 | 4.98 |
| KRILL | 14 | 75.41 | 2.80 | 17.87 | 1.36 | 4.94 |
| KRILL | 14 | 75.24 | 2.87 | 17.89 | 1.36 | 4.93 |
| KRILL | 21 | 76.10 | 2.83 | 17.36 | 1.25 | 4.78 |
| KRILL | 21 | 76.28 | 2.78 | 17.20 | 1.32 | 4.73 |
| KRILL | 25 | 75.32 | 2.82 | 17.81 | 1.43 | 4.99 |
| KRILL | 25 | 75.43 | 2.80 | 17.74 | 1.41 | 4.98 |
| TUNA | 1 | 76.01 | 2.93 | 17.59 | 1.07 | 4.93 |
| TUNA | 1 | 75.90 | 2.96 | 17.68 | 1.05 | 5.00 |
| TUNA | 2 | 77.34 | 3.07 | 16.56 | 0.91 | 4.49 |
| TUNA | 2 | 77.24 | 3.04 | 16.59 | 0.94 | 4.42 |
| TUNA | 6 | 77.16 | 2.90 | 16.69 | 1.08 | 4.41 |
| TUNA | 6 | 77.42 | 2.84 | 16.66 | 1.10 | 4.41 |
| TUNA | 10 | 76.60 | 2.98 | 17.05 | 0.96 | 4.56 |
| TUNA | 10 | 76.89 | 2.91 | 17.11 | 0.95 | 4.58 |
| TUNA | 16 | 76.07 | 3.06 | 17.14 | 1.01 | 4.64 |
| TUNA | 16 | 76.01 | 2.99 | 17.11 | 0.99 | 4.67 |

**Supplementary Table S5.** Nutrient retention of the shrimp after 62 days.

|  |  | **Retention (% intake)** | | |
| --- | --- | --- | --- | --- |
| **Diet** | **Tank** | **Protein** | **Lipid** | **Energy** |
| CTRL | 7 | 26.29 | 6.29 | 12.48 |
| CTRL | 11 | 26.62 | 6.96 | 13.05 |
| CTRL | 15 | 26.80 | 7.07 | 12.71 |
| CTRL | 17 | 26.95 | 6.83 | 12.88 |
| CTRL | 23 | 27.90 | 8.32 | 13.63 |
| CALANUS | 8 | 29.07 | 10.15 | 14.57 |
| CALANUS | 9 | 28.99 | 9.95 | 14.65 |
| CALANUS | 13 | 30.64 | 8.29 | 14.74 |
| CALANUS | 20 | 29.50 | 9.14 | 14.55 |
| CALANUS | 24 | 29.80 | 8.97 | 14.82 |
| SQUID | 3 | 28.04 | 6.70 | 13.67 |
| SQUID | 5 | 29.25 | 7.51 | 14.20 |
| SQUID | 18 | 29.03 | 7.18 | 13.92 |
| SQUID | 19 | 30.76 | 8.60 | 15.01 |
| SQUID | 22 | 28.69 | 7.91 | 13.74 |
| KRILL | 4 | 32.76 | 10.94 | 16.13 |
| KRILL | 12 | 30.12 | 9.98 | 14.80 |
| KRILL | 14 | 31.77 | 11.55 | 15.64 |
| KRILL | 21 | 29.06 | 10.33 | 14.26 |
| KRILL | 25 | 30.44 | 11.61 | 15.23 |
| TUNA | 1 | 26.20 | 7.37 | 13.17 |
| TUNA | 2 | 24.99 | 6.44 | 11.91 |
| TUNA | 6 | 26.24 | 8.08 | 12.26 |
| TUNA | 10 | 25.13 | 6.50 | 11.91 |
| TUNA | 16 | 27.31 | 7.42 | 13.17 |

**Supplementary Table S6.** Survival after salinity test.

|  |  |  | **g** |  |  | **%** |
| --- | --- | --- | --- | --- | --- | --- |
| **Diet** | **Tank** | **Initial** | **Initial** | **Dead** | **Final** | **Survival** |
|  |  | **Shrimp** | **Biomass** |  | **Shrimp** |  |
| CTRL | 7 | 61 | 895 | 9 | 52 | 85.2 |
| CTRL | 11 | 57 | 781 | 9 | 48 | 84.2 |
| CTRL | 15 | 56 | 784 | 8 | 48 | 85.7 |
| CTRL | 17 | 58 | 822 | 6 | 52 | 89.7 |
| CTRL | 23 | 56 | 827 | 7 | 49 | 87.5 |
| CALANUS | 8 | 56 | 819 | 6 | 50 | 89.3 |
| CALANUS | 9 | 60 | 866 | 5 | 55 | 91.7 |
| CALANUS | 13 | 61 | 889 | 6 | 55 | 90.2 |
| CALANUS | 20 | 59 | 872 | 6 | 53 | 89.8 |
| CALANUS | 24 | 58 | 861 | 7 | 51 | 87.9 |
| SQUID | 3 | 63 | 904 | 7 | 56 | 88.9 |
| SQUID | 5 | 60 | 853 | 7 | 53 | 88.3 |
| SQUID | 18 | 55 | 832 | 9 | 46 | 83.6 |
| SQUID | 19 | 57 | 858 | 7 | 50 | 87.7 |
| SQUID | 22 | 58 | 827 | 8 | 50 | 86.2 |
| KRILL | 4 | 57 | 833 | 6 | 51 | 89.5 |
| KRILL | 12 | 54 | 766 | 6 | 48 | 88.9 |
| KRILL | 14 | 56 | 783 | 8 | 48 | 85.7 |
| KRILL | 21 | 59 | 837 | 7 | 52 | 88.1 |
| KRILL | 25 | 59 | 810 | 7 | 52 | 88.1 |
| TUNA | 1 | 54 | 647 | 8 | 46 | 85.2 |
| TUNA | 2 | 55 | 716 | 8 | 47 | 85.5 |
| TUNA | 6 | 53 | 662 | 10 | 43 | 81.1 |
| TUNA | 10 | 52 | 667 | 7 | 45 | 86.5 |
| TUNA | 16 | 56 | 745 | 9 | 47 | 83.9 |

**Supplementary Table S7.** Immune criteria after the salinity test.

|  |  |  | **U/mg protein** | **µg/ml** | **%** |
| --- | --- | --- | --- | --- | --- |
|  | **Tank** | **Fish #** | **Prophenoloxidase** | **Lysozyme** | **Bactericidal activity** |
| CTRL | 7 | 1 | 0.199 | 0.90 | 25.86 |
| CTRL | 7 | 2 | 0.215 |  | 22.42 |
| CTRL | 11 | 1 | 0.240 | 1.19 | 27.67 |
| CTRL | 11 | 2 | 0.175 | 1.15 | 24.39 |
| CTRL | 15 | 1 | 0.244 | 0.89 | 24.68 |
| CTRL | 15 | 2 | 0.239 | 0.79 | 22.29 |
| CTRL | 17 | 1 | 0.224 | 0.76 | 24.54 |
| CTRL | 17 | 2 | 0.219 | 0.81 |  |
| CTRL | 23 | 1 | 0.205 | 0.83 | 27.88 |
| CTRL | 23 | 2 | 0.235 | 0.62 | 35.22 |
| CALANUS | 8 | 1 | 0.366 | 1.05 | 34.03 |
| CALANUS | 8 | 2 | 0.395 | 1.13 | 43.12 |
| CALANUS | 9 | 1 | 0.494 | 1.15 | 36.90 |
| CALANUS | 9 | 2 |  | 1.14 | 35.08 |
| CALANUS | 13 | 1 | 0.413 | 1.06 | 32.29 |
| CALANUS | 13 | 2 | 0.382 | 1.09 | 41.94 |
| CALANUS | 20 | 1 | 0.466 | 1.07 | 33.21 |
| CALANUS | 20 | 2 | 0.483 | 1.07 | 32.43 |
| CALANUS | 24 | 1 | 0.473 | 1.06 | 27.61 |
| CALANUS | 24 | 2 | 0.491 | 1.05 | 32.52 |
| SQUID | 3 | 1 | 0.446 | 0.80 | 37.67 |
| SQUID | 3 | 2 | 0.481 |  | 32.75 |
| SQUID | 5 | 1 | 0.316 | 0.47 | 32.84 |
| SQUID | 5 | 2 | 0.366 | 0.70 | 30.96 |
| SQUID | 18 | 1 | 0.352 | 1.02 | 30.18 |
| SQUID | 18 | 2 | 0.426 | 1.02 |  |
| SQUID | 19 | 1 | 0.374 | 0.78 | 30.12 |
| SQUID | 19 | 2 | 0.410 | 0.93 |  |
| SQUID | 22 | 1 | 0.417 | 0.97 | 33.69 |
| SQUID | 22 | 2 | 0.357 | 1.09 | 33.90 |
| KRILL | 4 | 1 |  | 1.16 | 33.26 |
| KRILL | 4 | 2 | 0.578 | 1.19 | 30.61 |
| KRILL | 12 | 1 | 0.610 | 1.04 | 37.53 |
| KRILL | 12 | 2 | 0.545 | 0.83 | 25.09 |
| KRILL | 14 | 1 | 0.467 | 1.07 |  |
| KRILL | 14 | 2 | 0.498 | 1.07 | 28.93 |
| KRILL | 21 | 1 | 0.518 | 1.19 |  |
| KRILL | 21 | 2 | 0.519 | 1.16 | 32.49 |
| KRILL | 25 | 1 | 0.499 | 1.07 |  |
| KRILL | 25 | 2 | 0.521 | 1.09 |  |
| TUNA | 1 | 1 | 0.355 | 1.12 | 29.76 |
| TUNA | 1 | 2 | 0.322 | 1.10 | 18.45 |
| TUNA | 2 | 1 | 0.374 | 1.03 | 29.48 |
| TUNA | 2 | 2 | 0.344 | 1.13 | 33.54 |
| TUNA | 6 | 1 | 0.298 | 1.26 | 22.91 |
| TUNA | 6 | 2 | 0.309 | 0.93 | 23.67 |
| TUNA | 10 | 1 |  | 0.86 | 25.29 |
| TUNA | 10 | 2 | 0.360 | 0.86 | 31.94 |
| TUNA | 16 | 1 | 0.346 | 0.78 | 28.13 |
| TUNA | 16 | 2 | 0.385 | 0.89 |  |

**Supplementary Table S8.** Oxidative stress parameters after the salinity test.

|  |  |  | **mU/mg prot** | **mU/mg prot** | **μM/mg prot** | **nmol/mg tissue** | **nmol MDA/mg tissue** |
| --- | --- | --- | --- | --- | --- | --- | --- |
|  | **Tank** | **Fish #** | **SOD** | **CAT** | **tGSH** | **PC** | **LPO** |
| CTRL | 7 | 1 | 12.62 | 44.62 | 7.22 | 18.55 | 39.73 |
| CTRL | 7 | 2 | 14.28 | 40.52 | 7.83 | 17.91 | 38.16 |
| CTRL | 11 | 1 | 16.53 | 55.27 | 6.90 | 27.84 | 44.28 |
| CTRL | 11 | 2 | 16.81 | 50.11 | 6.51 | 25.12 | 40.45 |
| CTRL | 15 | 1 | 9.35 | 44.61 | 8.25 | 22.91 | 37.73 |
| CTRL | 15 | 2 | 12.15 | 46.27 | 7.86 | 20.37 | 40.48 |
| CTRL | 17 | 1 | 14.27 | 39.63 | 5.73 | 16.92 | 34.71 |
| CTRL | 17 | 2 | 13.76 | 42.43 | 5.19 | 16.24 | 29.94 |
| CTRL | 23 | 1 | 12.58 | 49.46 | 12.61 | 25.53 | 32.29 |
| CTRL | 23 | 2 | 14.33 | 44.18 | 9.17 | 20.38 | 30.47 |
| CALANUS | 8 | 1 | 14.78 | 52.57 | 17.25 | 18.62 | 31.27 |
| CALANUS | 8 | 2 | 17.84 | 49.27 | 16.72 | 19.42 | 30.64 |
| CALANUS | 9 | 1 | 17.21 | 51.24 | 12.12 | 23.44 | 27.64 |
| CALANUS | 9 | 2 | 19.12 | 52.81 | 13.63 | 21.37 | 26.88 |
| CALANUS | 13 | 1 | 18.66 | 43.62 | 10.94 | 23.12 | 30.81 |
| CALANUS | 13 | 2 | 17.72 | 49.71 | 13.82 | 19.52 | 29.73 |
| CALANUS | 20 | 1 | 19.72 | 39.97 | 18.83 | 21.66 | 35.17 |
| CALANUS | 20 | 2 | 21.16 | 43.94 | 17.24 | 19.38 | 31.66 |
| CALANUS | 24 | 1 | 19.64 | 44.70 | 17.27 | 19.82 | 27.33 |
| CALANUS | 24 | 2 | 20.17 | 45.24 | 16.48 | 21.47 | 26.96 |
| SQUID | 3 | 1 | 14.73 | 48.13 | 9.18 | 17.18 | 42.44 |
| SQUID | 3 | 2 | 16.32 | 43.97 | 10.61 | 16.82 | 39.52 |
| SQUID | 5 | 1 | 9.29 | 47.06 | 14.11 | 24.87 | 30.47 |
| SQUID | 5 | 2 | 11.24 | 50.16 | 12.73 | 23.66 | 34.22 |
| SQUID | 18 | 1 | 16.34 | 39.74 | 9.85 | 21.84 | 31.49 |
| SQUID | 18 | 2 | 13.92 | 45.82 | 10.14 | 20.71 | 36,73 |
| SQUID | 19 | 1 | 17.78 | 41.63 | 11.16 | 20.08 | 37.73 |
| SQUID | 19 | 2 | 18.27 | 44.36 | 13.84 | 24.49 | 34.56 |
| SQUID | 22 | 1 | 15.36 | 47.29 | 12.64 | 28.72 | 37.74 |
| SQUID | 22 | 2 | 12.51 | 46.18 | 9.87 | 24.15 | 38.03 |
| KRILL | 4 | 1 | 23.84 | 51.12 | 16.77 | 17.54 | 32.13 |
| KRILL | 4 | 2 | 24.91 | 52.72 | 21.62 | 20.27 | 29.31 |
| KRILL | 12 | 1 | 19.22 | 39.48 | 19.11 | 19.63 | 25.30 |
| KRILL | 12 | 2 | 18.67 | 43.64 | 20.03 | 18.73 | 26.47 |
| KRILL | 14 | 1 | 14.37 | 46.25 | 24.62 | 17.41 | 23.62 |
| KRILL | 14 | 2 | 19.84 | 43.16 | 22.13 | 15.19 | 23.77 |
| KRILL | 21 | 1 | 18.57 | 54.12 | 20.38 | 19.16 | 28.41 |
| KRILL | 21 | 2 | 15.72 | 50.18 | 22.17 | 20.04 | 30.39 |
| KRILL | 25 | 1 | 22.11 | 47.71 | 22.84 | 23.53 | 26.02 |
| KRILL | 25 | 2 | 16.83 | 56.21 | 19.76 | 25.21 | 28.14 |
| TUNA | 1 | 1 | 16.28 | 42.63 | 21.48 | 20.33 | 31.69 |
| TUNA | 1 | 2 | 12.97 | 49.38 | 18.73 | 22.93 | 37.36 |
| TUNA | 2 | 1 | 16.48 | 46.89 | 15.95 | 20.80 | 33.64 |
| TUNA | 2 | 2 | 20.43 | 51.17 | 18.38 | 22.54 | 37.19 |
| TUNA | 6 | 1 | 20.72 | 51.28 | 21.27 | 20.72 | 36.72 |
| TUNA | 6 | 2 | 21.14 | 47.92 | 10.64 | 23.64 | 30.07 |
| TUNA | 10 | 1 | 19.12 | 46.26 | 17.52 | 18.29 | 31.05 |
| TUNA | 10 | 2 | 18.42 | 50.11 | 15.88 | 23.52 | 34.27 |
| TUNA | 16 | 1 | 14.46 | 50.38 | 15.17 | 20.34 | 30.48 |
| TUNA | 16 | 2 | 16.83 | 57.31 | 16.82 | 21.13 | 32.83 |

**Supplementary Table S9.** Metabolic status after the salinity test.

|  |  | **mU/mg protein** | **mU/mg protein** |
| --- | --- | --- | --- |
|  | **Tank** | **ALT** | **AST** |
| CTRL | 7 | 22.76 | 23.85 |
| CTRL | 11 | 24.26 | 27.70 |
| CTRL | 15 | 24.92 | 34.20 |
| CTRL | 17 | 25.00 | 26.66 |
| CTRL | 23 | 22.71 | 26.87 |
| CALANUS | 8 | 18.23 | 12.19 |
| CALANUS | 9 | 19.19 | 18.93 |
| CALANUS | 13 | 20.78 | 17.89 |
| CALANUS | 20 | 19.06 | 17.25 |
| CALANUS | 24 | 18.68 | 12.68 |
| SQUID | 3 | 23.81 | 16.38 |
| SQUID | 5 | 22.36 | 19.01 |
| SQUID | 18 | 23.62 | 26.28 |
| SQUID | 19 | 21.69 | 25.52 |
| SQUID | 22 | 22.82 | 25.01 |
| KRILL | 4 | 16.47 | 13.99 |
| KRILL | 12 | 20.49 | 9.39 |
| KRILL | 14 | 18.20 | 8.87 |
| KRILL | 21 | 18.86 | 15.16 |
| KRILL | 25 | 19.35 | 17.40 |
| TUNA | 1 | 22.66 | 19.02 |
| TUNA | 2 | 23.46 | 13.67 |
| TUNA | 6 | 24.12 | 19.94 |
| TUNA | 10 | 22.75 | 19.65 |
| TUNA | 16 | 23.08 | 25.11 |

| **mg/g DM** | **Tank** | **Arg** | **His** | **Ile** | **Leu** | **Lys** | **Thr** | **Trp** | **Val** | **Met** | **Cys** | **Phe** | **Tyr** |
| --- | --- | --- | --- | --- | --- | --- | --- | --- | --- | --- | --- | --- | --- |
| CTRL | 7 | 23.69 | 7.83 | 4.50 | 14.19 | 14.03 | 13.85 | 3.69 | 3.55 | 8.27 | 0.12 | 11.95 | 5.48 |
| CTRL | 11 | 21.48 | 7.05 | 5.52 | 12.99 | 14.24 | 10.52 | 2.66 | 2.70 | 7.47 | 0.14 | 11.91 | 5.50 |
| CTRL | 15 | 22.67 | 7.12 | 3.91 | 12.86 | 13.68 | 13.35 | 2.60 | 2.62 | 7.23 | 0.10 | 11.58 | 5.32 |
| CTRL | 17 | 22.93 | 6.80 | 4.41 | 10.70 | 13.54 | 12.31 | 2.28 | 2.92 | 6.07 | 0.13 | 11.32 | 5.29 |
| CTRL | 23 | 20.34 | 6.79 | 4.38 | 12.75 | 13.32 | 13.23 | 2.53 | 2.51 | 7.08 | 0.11 | 11.31 | 5.19 |
| CALANUS | 8 | 25.55 | 8.10 | 5.57 | 16.92 | 13.88 | 8.19 | 3.65 | 4.84 | 7.90 | 0.15 | 16.35 | 6.65 |
| CALANUS | 9 | 21.87 | 7.33 | 6.20 | 15.84 | 12.63 | 7.50 | 3.34 | 5.45 | 7.25 | 0.15 | 14.67 | 5.70 |
| CALANUS | 13 | 24.58 | 6.10 | 7.83 | 15.73 | 13.24 | 6.69 | 3.33 | 5.79 | 8.31 | 0.17 | 14.89 | 6.39 |
| CALANUS | 20 | 23.00 | 6.71 | 6.08 | 15.05 | 13.14 | 7.34 | 3.93 | 5.72 | 7.87 | 0.19 | 13.74 | 6.22 |
| CALANUS | 24 | 23.87 | 7.35 | 5.03 | 15.76 | 12.77 | 7.47 | 3.22 | 4.79 | 7.84 | 0.19 | 14.69 | 6.76 |
| SQUID | 3 | 12.20 | 10.19 | 6.13 | 14.34 | 14.90 | 6.88 | 2.95 | 4.72 | 7.38 | 0.11 | 16.14 | 6.25 |
| SQUID | 5 | 15.02 | 12.06 | 5.24 | 16.37 | 13.73 | 7.79 | 2.74 | 5.69 | 7.07 | 0.14 | 14.11 | 6.16 |
| SQUID | 18 | 11.88 | 8.06 | 5.07 | 17.01 | 14.43 | 6.43 | 3.17 | 4.20 | 7.02 | 0.14 | 11.78 | 6.00 |
| SQUID | 19 | 11.73 | 10.39 | 5.08 | 16.49 | 13.88 | 6.32 | 3.77 | 5.37 | 6.12 | 0.11 | 13.17 | 6.18 |
| SQUID | 22 | 13.47 | 11.06 | 5.21 | 16.52 | 14.76 | 6.06 | 3.08 | 5.69 | 6.16 | 0.15 | 13.83 | 6.17 |
| KRILL | 4 | 19.08 | 8.06 | 4.96 | 15.19 | 13.63 | 6.80 | 2.95 | 3.52 | 5.47 | 0.22 | 14.26 | 6.23 |
| KRILL | 12 | 16.28 | 5.95 | 5.05 | 15.59 | 13.95 | 4.79 | 3.48 | 3.61 | 5.62 | 0.21 | 13.60 | 6.44 |
| KRILL | 14 | 19.34 | 5.97 | 5.07 | 15.62 | 13.92 | 3.80 | 3.15 | 4.68 | 8.63 | 0.19 | 13.64 | 6.42 |
| KRILL | 21 | 20.05 | 6.18 | 5.98 | 15.73 | 13.94 | 4.79 | 3.05 | 3.63 | 6.67 | 0.18 | 12.64 | 6.40 |
| KRILL | 25 | 18.90 | 7.76 | 4.98 | 15.33 | 13.68 | 4.77 | 2.69 | 3.07 | 6.51 | 0.17 | 12.37 | 6.27 |
| TUNA | 1 | 13.12 | 8.15 | 4.56 | 15.68 | 13.46 | 5.39 | 3.19 | 4.48 | 6.97 | 0.13 | 12.68 | 10.15 |
| TUNA | 2 | 12.95 | 8.08 | 4.63 | 14.78 | 13.64 | 4.28 | 3.85 | 4.42 | 8.98 | 0.10 | 13.71 | 9.88 |
| TUNA | 6 | 14.49 | 7.13 | 4.68 | 16.06 | 13.81 | 5.42 | 3.72 | 3.55 | 9.13 | 0.11 | 13.85 | 10.29 |
| TUNA | 10 | 11.62 | 7.57 | 5.43 | 15.43 | 14.05 | 4.12 | 3.70 | 3.43 | 8.66 | 0.09 | 15.12 | 9.80 |
| TUNA | 16 | 13.42 | 9.19 | 5.66 | 14.07 | 12.78 | 6.30 | 4.25 | 3.19 | 7.08 | 0.09 | 13.82 | 10.20 |

| **mg/g DM** | **Tank** | **Asp** | **Glu** | **Ala** | **Gly** | **Pro** | **Ser** |
| --- | --- | --- | --- | --- | --- | --- | --- |
| CTRL | 7 | 4.91 | 20.99 | 15.08 | 11.72 | 11.98 | 16.93 |
| CTRL | 11 | 4.69 | 20.95 | 14.99 | 12.57 | 13.75 | 16.79 |
| CTRL | 15 | 4.58 | 21.25 | 14.29 | 9.57 | 11.17 | 16.35 |
| CTRL | 17 | 4.62 | 19.98 | 14.45 | 11.48 | 12.16 | 16.26 |
| CTRL | 23 | 4.48 | 18.80 | 14.15 | 13.45 | 10.78 | 15.97 |
| CALANUS | 8 | 5.17 | 12.70 | 7.90 | 9.64 | 9.29 | 17.06 |
| CALANUS | 9 | 4.96 | 11.09 | 9.52 | 8.56 | 8.40 | 16.15 |
| CALANUS | 13 | 5.15 | 13.49 | 7.54 | 8.53 | 8.29 | 15.38 |
| CALANUS | 20 | 4.86 | 12.09 | 8.50 | 9.07 | 8.01 | 14.89 |
| CALANUS | 24 | 4.71 | 14.20 | 8.21 | 8.62 | 9.45 | 17.34 |
| SQUID | 3 | 5.06 | 11.37 | 9.64 | 13.29 | 12.25 | 18.43 |
| SQUID | 5 | 4.04 | 9.31 | 9.60 | 11.19 | 14.13 | 18.06 |
| SQUID | 18 | 3.95 | 9.09 | 9.28 | 10.78 | 12.80 | 17.65 |
| SQUID | 19 | 4.09 | 10.37 | 8.73 | 9.21 | 11.26 | 18.26 |
| SQUID | 22 | 4.04 | 9.32 | 8.67 | 11.04 | 10.11 | 16.85 |
| KRILL | 4 | 4.66 | 11.87 | 8.31 | 7.17 | 7.91 | 17.41 |
| KRILL | 12 | 5.64 | 14.19 | 8.39 | 6.32 | 8.94 | 17.37 |
| KRILL | 14 | 3.84 | 12.29 | 10.54 | 9.32 | 7.29 | 16.38 |
| KRILL | 21 | 4.61 | 12.46 | 8.42 | 7.46 | 7.14 | 15.39 |
| KRILL | 25 | 5.11 | 10.85 | 7.24 | 6.27 | 8.10 | 16.97 |
| TUNA | 1 | 6.20 | 20.24 | 14.97 | 14.96 | 11.14 | 14.51 |
| TUNA | 2 | 5.14 | 18.32 | 14.92 | 16.01 | 13.84 | 14.34 |
| TUNA | 6 | 4.16 | 19.73 | 15.32 | 15.08 | 12.28 | 14.58 |
| TUNA | 10 | 3.94 | 20.68 | 14.42 | 14.82 | 11.18 | 13.96 |
| TUNA | 16 | 5.21 | 17.79 | 12.17 | 16.05 | 12.07 | 14.60 |

| **mg/g DM** | **Tank** | **Asn** | **Gln** | **Tau** | **Orn** | **GABA** | **HPro** | **BAla** | **HCys** | **Cysta** | **TMG** | **SAM** | **SAH** |
| --- | --- | --- | --- | --- | --- | --- | --- | --- | --- | --- | --- | --- | --- |
| CTRL | 7 | 2.55 | 13.90 | 5.26 | 2.88 | 0.47 | 0.19 | 0.23 | 0.0262 | 0.0486 | 0.0333 | 1.08 | 0.18 |
| CTRL | 11 | 2.54 | 12.74 | 5.28 | 2.84 | 0.42 | 0.22 | 0.23 | 0.0262 | 0.0508 | 0.0328 | 1.15 | 0.19 |
| CTRL | 15 | 2.49 | 12.06 | 6.16 | 2.83 | 0.36 | 0.17 | 0.22 | 0.0246 | 0.0490 | 0.0325 | 1.13 | 0.18 |
| CTRL | 17 | 2.45 | 13.42 | 6.14 | 2.76 | 0.42 | 0.22 | 0.21 | 0.0247 | 0.0472 | 0.0325 | 1.05 | 0.18 |
| CTRL | 23 | 2.42 | 11.94 | 5.00 | 2.90 | 0.38 | 0.16 | 0.21 | 0.0244 | 0.0463 | 0.0312 | 1.08 | 0.19 |
| CALANUS | 8 | 2.61 | 12.97 | 3.16 | 3.44 | 0.64 | 0.31 | 0.28 | 0.0269 | 0.0764 | 0.0677 | 1.42 | 0.15 |
| CALANUS | 9 | 2.15 | 11.93 | 3.92 | 3.51 | 0.74 | 0.47 | 0.25 | 0.0273 | 0.0761 | 0.0637 | 1.36 | 0.21 |
| CALANUS | 13 | 2.38 | 10.58 | 3.64 | 3.64 | 0.67 | 0.43 | 0.24 | 0.0251 | 0.0756 | 0.0648 | 1.42 | 0.18 |
| CALANUS | 20 | 1.71 | 11.29 | 3.59 | 3.57 | 0.72 | 0.41 | 0.24 | 0.0290 | 0.0739 | 0.0619 | 1.36 | 0.14 |
| CALANUS | 24 | 2.15 | 11.54 | 3.53 | 3.94 | 0.74 | 0.38 | 0.25 | 0.0258 | 0.0761 | 0.0608 | 1.39 | 0.20 |
| SQUID | 3 | 3.97 | 10.83 | 6.54 | 3.38 | 0.75 | 0.39 | 0.28 | 0.0257 | 0.0560 | 0.0310 | 1.02 | 0.18 |
| SQUID | 5 | 4.21 | 12.77 | 7.40 | 3.34 | 0.66 | 0.38 | 0.28 | 0.0256 | 0.0565 | 0.0409 | 1.12 | 0.16 |
| SQUID | 18 | 4.02 | 10.54 | 9.29 | 3.34 | 0.73 | 0.35 | 0.27 | 0.0254 | 0.0549 | 0.0395 | 1.06 | 0.14 |
| SQUID | 19 | 4.22 | 10.52 | 9.67 | 3.33 | 0.73 | 0.35 | 0.28 | 0.0258 | 0.0565 | 0.0398 | 1.08 | 0.16 |
| SQUID | 22 | 4.16 | 8.77 | 8.39 | 3.34 | 0.64 | 0.39 | 0.27 | 0.0257 | 0.0555 | 0.0352 | 1.07 | 0.14 |
| KRILL | 4 | 2.17 | 12.67 | 5.06 | 2.95 | 0.78 | 0.15 | 0.26 | 0.0269 | 0.0463 | 0.0625 | 1.35 | 0.17 |
| KRILL | 12 | 2.05 | 12.89 | 4.06 | 2.99 | 0.69 | 0.10 | 0.19 | 0.0253 | 0.0490 | 0.0591 | 1.20 | 0.15 |
| KRILL | 14 | 2.34 | 9.69 | 4.13 | 2.84 | 0.76 | 0.11 | 0.22 | 0.0241 | 0.0582 | 0.0614 | 1.15 | 0.17 |
| KRILL | 21 | 2.30 | 8.54 | 3.21 | 2.88 | 0.68 | 0.12 | 0.20 | 0.0269 | 0.0587 | 0.0575 | 1.29 | 0.17 |
| KRILL | 25 | 2.23 | 9.41 | 3.96 | 2.84 | 0.78 | 0.12 | 0.19 | 0.0262 | 0.0582 | 0.0573 | 1.21 | 0.16 |
| TUNA | 1 | 2.49 | 12.36 | 6.50 | 2.99 | 0.69 | 0.25 | 0.24 | 0.0269 | 0.0466 | 0.0338 | 1.12 | 0.19 |
| TUNA | 2 | 2.30 | 11.45 | 5.34 | 2.83 | 0.69 | 0.22 | 0.21 | 0.0257 | 0.0476 | 0.0337 | 1.13 | 0.18 |
| TUNA | 6 | 2.43 | 12.65 | 4.56 | 3.11 | 0.71 | 0.22 | 0.25 | 0.0262 | 0.0487 | 0.0335 | 1.09 | 0.17 |
| TUNA | 10 | 2.34 | 12.04 | 5.21 | 2.80 | 0.67 | 0.18 | 0.23 | 0.0247 | 0.0458 | 0.0317 | 1.07 | 0.22 |
| TUNA | 16 | 2.21 | 13.53 | 5.42 | 2.92 | 0.68 | 0.20 | 0.27 | 0.0254 | 0.0477 | 0.0333 | 1.10 | 0.15 |

**Supplementary Table S10.** Statistical analyses (ANOVA, Brown-Forsythe, Tukey post-hoc).

Supplementary Table S10.1. Summary of one-way ANOVA growth performance.

| **Table Analyzed** |  |  |  |  |
| --- | --- | --- | --- | --- |
|  |  |  |  |  |
| **ANOVA summary** | **Body weight** | **SGR** | **FCR** | **PER** |
| F | 19,36 | 19,59 | 21,87 | 20,35 |
| P value | <0,0001 | <0,0001 | <0,0001 | <0,0001 |
| P value summary | **** | **** | **** | **** |
| Significant diff. among means (P < 0.05)? | Yes | Yes | Yes | Yes |
| R squared | 0,7947 | 0,7967 | 0,8139 | 0,8027 |
|  |  |  |  |  |
| **Brown-Forsythe test** |  |  |  |  |
| F (DFn, DFd) | 0,7093 (4, 20) | 0,7350 (4, 20) | 0,9013 (4, 20) | 0,6149 (4, 20) |
| P value | 0,5951 | 0,5789 | 0,4818 | 0,6569 |
| P value summary | ns | ns | ns | ns |
| Are SDs significantly different (P < 0.05)? | No | No | No | No |
|  |  |  |  |  |
| **Data summary** |  |  |  |  |
| Number of treatments (columns) | 5 | 5 | 5 | 5 |
| Number of values (total) | 25 | 25 | 25 | 25 |

| **ANOVA table Body weight** | **SS** | **DF** | **MS** | **F (DFn, DFd)** | **P value** |
| --- | --- | --- | --- | --- | --- |
| Treatment (between columns) | 13,34 | 4 | 3,336 | F (4, 20) = 19,36 | P<0,0001 |
| Residual (within columns) | 3,447 | 20 | 0,1724 |  |  |
| Total | 16,79 | 24 |  |  |  |
|  |  |  |  |  |  |
| **ANOVA table SGR** | **SS** | **DF** | **MS** | **F (DFn, DFd)** | **P value** |
| Treatment (between columns) | 0,1923 | 4 | 0,04808 | F (4, 20) = 19,59 | P<0,0001 |
| Residual (within columns) | 0,04908 | 20 | 0,002454 |  |  |
| Total | 0,2414 | 24 |  |  |  |
|  |  |  |  |  |  |
| **ANOVA table FCR** | **SS** | **DF** | **MS** | **F (DFn, DFd)** | **P value** |
| Treatment (between columns) | 0,3791 | 4 | 0,09478 | F (4, 20) = 21,87 | P<0,0001 |
| Residual (within columns) | 0,08668 | 20 | 0,004334 |  |  |
| Total | 0,4658 | 24 |  |  |  |
|  |  |  |  |  |  |
| **ANOVA table PER** | **SS** | **DF** | **MS** | **F (DFn, DFd)** | **P value** |
| Treatment (between columns) | 0,2222 | 4 | 0,05555 | F (4, 20) = 20,35 | P<0,0001 |
| Residual (within columns) | 0,05460 | 20 | 0,002730 |  |  |
| Total | 0,2768 | 24 |  |  |  |

Supplementary Table S10.2. Summary of one-way ANOVA whole-body composition.

| **Table Analyzed** |  |  |  |  |  |
| --- | --- | --- | --- | --- | --- |
|  |  |  |  |  |  |
| **ANOVA summary** | **Moisture** | **Ash** | **Protein** | **Fat** | **Energy** |
| F | 3,452 | 3,282 | 4,185 | 15,84 | 3,156 |
| P value | 0,0267 | 0,0319 | 0,0127 | <0,0001 | 0,0365 |
| P value summary | * | * | * | **** | * |
| Significant diff. among means (P < 0.05)? | Yes | Yes | Yes | Yes | Yes |
| R squared | 0,4084 | 0,3963 | 0,4556 | 0,7601 | 0,3870 |
|  |  |  |  |  |  |
| **Brown-Forsythe test** |  |  |  |  |  |
| F (DFn, DFd) | 1,070 (4, 20) | 0,7247 (4, 20) | 0,7716 (4, 20) | 0,4006 (4, 20) | 0,5683 (4, 20) |
| P value | 0,3974 | 0,5853 | 0,5564 | 0,8059 | 0,6885 |
| P value summary | ns | ns | ns | ns | ns |
| Are SDs significantly different (P < 0.05)? | No | No | No | No | No |
|  |  |  |  |  |  |
| **Data summary** |  |  |  |  |  |
| Number of treatments (columns) | 5 | 5 | 5 | 5 | 5 |
| Number of values (total) | 25 | 25 | 25 | 25 | 25 |

| **ANOVA table Moisture** | **SS** | **DF** | **MS** | **F (DFn, DFd)** | **P value** |
| --- | --- | --- | --- | --- | --- |
| Treatment (between columns) | 3,548 | 4 | 0,8870 | F (4, 20) = 3,452 | P=0,0267 |
| Residual (within columns) | 5,140 | 20 | 0,2570 |  |  |
| Total | 8,688 | 24 |  |  |  |
|  |  |  |  |  |  |
| **ANOVA table Ash** | **SS** | **DF** | **MS** | **F (DFn, DFd)** | **P value** |
| Treatment (between columns) | 0,1558 | 4 | 0,03895 | F (4, 20) = 3,282 | P=0,0319 |
| Residual (within columns) | 0,2374 | 20 | 0,01187 |  |  |
| Total | 0,3932 | 24 |  |  |  |
|  |  |  |  |  |  |
| **ANOVA table Protein** | **SS** | **DF** | **MS** | **F (DFn, DFd)** | **P value** |
| Treatment (between columns) | 2,036 | 4 | 0,5091 | F (4, 20) = 4,185 | P=0,0127 |
| Residual (within columns) | 2,433 | 20 | 0,1217 |  |  |
| Total | 4,469 | 24 |  |  |  |
|  |  |  |  |  |  |
| **ANOVA table Fat** | **SS** | **DF** | **MS** | **F (DFn, DFd)** | **P value** |
| Treatment (between columns) | 0,4491 | 4 | 0,1123 | F (4, 20) = 15,84 | P<0,0001 |
| Residual (within columns) | 0,1417 | 20 | 0,007086 |  |  |
| Total | 0,5908 | 24 |  |  |  |
|  |  |  |  |  |  |
| **ANOVA table Energy** | **SS** | **DF** | **MS** | **F (DFn, DFd)** | **P value** |
| Treatment (between columns) | 0,2731 | 4 | 0,06828 | F (4, 20) = 3,156 | P=0,0365 |
| Residual (within columns) | 0,4327 | 20 | 0,02163 |  |  |
| Total | 0,7058 | 24 |  |  |  |

Supplementary Table S10.3. Summary of one-way ANOVA nutrient retention.

| **Table Analyzed** |  |  |  |
| --- | --- | --- | --- |
|  |  |  |  |
| **ANOVA summary** | **Protein** | **Lipid** | **Energy** |
| F | 20,69 | 25,58 | 22,88 |
| P value | <0,0001 | <0,0001 | <0,0001 |
| P value summary | **** | **** | **** |
| Significant diff. among means (P < 0.05)? | Yes | Yes | Yes |
| R squared | 0,8054 | 0,8365 | 0,8206 |
|  |  |  |  |
| **Brown-Forsythe test** |  |  |  |
| F (DFn, DFd) | 0,8247 (4, 20) | 0,05471 (4, 20) | 1,412 (4, 20) |
| P value | 0,5249 | 0,9940 | 0,2660 |
| P value summary | ns | ns | ns |
| Are SDs significantly different (P < 0.05)? | No | No | No |
|  |  |  |  |
| **Data summary** |  |  |  |
| Number of treatments (columns) | 5 | 5 | 5 |
| Number of values (total) | 25 | 25 | 25 |

| **ANOVA table Protein** | **SS** | **DF** | **MS** | **F (DFn, DFd)** | **P value** |
| --- | --- | --- | --- | --- | --- |
| Treatment (between columns) | 79,84 | 4 | 19,96 | F (4, 20) = 20,69 | P<0,0001 |
| Residual (within columns) | 19,29 | 20 | 0,9646 |  |  |
| Total | 99,14 | 24 |  |  |  |
|  |  |  |  |  |  |
| **ANOVA table Lipid** | **SS** | **DF** | **MS** | **F (DFn, DFd)** | **P value** |
| Treatment (between columns) | 54,40 | 4 | 13,60 | F (4, 20) = 25,58 | P<0,0001 |
| Residual (within columns) | 10,64 | 20 | 0,5318 |  |  |
| Total | 65,04 | 24 |  |  |  |
|  |  |  |  |  |  |
| **ANOVA table Energy** | **SS** | **DF** | **MS** | **F (DFn, DFd)** | **P value** |
| Treatment (between columns) | 26,29 | 4 | 6,572 | F (4, 20) = 22,88 | P<0,0001 |
| Residual (within columns) | 5,745 | 20 | 0,2873 |  |  |
| Total | 32,03 | 24 |  |  |  |

Supplementary Table S10.4. Summary of one-way ANOVA survival.

| **Table Analyzed** |  |  |
| --- | --- | --- |
|  |  |  |
| **ANOVA summary** | Pre-salinity | Post-salinity |
| F | 3,826 | 5,559 |
| P value | 0,0182 | 0,0036 |
| P value summary | * | ** |
| Significant diff. among means (P < 0.05)? | Yes | Yes |
| R squared | 0,4335 | 0,5265 |
|  |  |  |
| **Brown-Forsythe test** |  |  |
| F (DFn, DFd) | 0,3841 (4, 20) | 0,2520 (4, 20) |
| P value | 0,8174 | 0,9050 |
| P value summary | ns | ns |
| Are SDs significantly different (P < 0.05)? | No | No |
|  |  |  |
| **Data summary** |  |  |
| Number of treatments (columns) | 5 | 5 |
| Number of values (total) | 25 | 25 |

| **ANOVA table Pre-salinity challenge** | **SS** | **DF** | **MS** | **F (DFn, DFd)** | **P value** |
| --- | --- | --- | --- | --- | --- |
| Treatment (between columns) | 117,0 | 4 | 29,26 | F (4, 20) = 3,826 | P=0,0182 |
| Residual (within columns) | 152,9 | 20 | 7,647 |  |  |
| Total | 270,0 | 24 |  |  |  |
|  |  |  |  |  |  |
| **ANOVA table Post-salinity challenge** | **SS** | **DF** | **MS** | **F (DFn, DFd)** | **P value** |
| Treatment (between columns) | 78,04 | 4 | 19,51 | F (4, 20) = 5,559 | P=0,0036 |
| Residual (within columns) | 70,20 | 20 | 3,510 |  |  |
| Total | 148,2 | 24 |  |  |  |

Supplementary Table S10.5. Summary of one-way ANOVA immune criteria.

| **Table Analyzed** |  |  |  |
| --- | --- | --- | --- |
|  |  |  |  |
| **ANOVA summary** | **Prophenoloxidase** | **Lysozyme** | **Bactericidal capacity** |
| F | 46,50 | 3,260 | 7,923 |
| P value | <0,0001 | 0,0327 | 0,0006 |
| P value summary | **** | * | *** |
| Significant diff. among means (P < 0.05)? | Yes | Yes | Yes |
| R squared | 0,9029 | 0,3947 | 0,6252 |
|  |  |  |  |
| **Brown-Forsythe test** |  |  |  |
| F (DFn, DFd) | 0,8088 (4, 20) | 0,9907 (4, 20) | 0,4107 (4, 19) |
| P value | 0,5341 | 0,4353 | 0,7987 |
| P value summary | ns | ns | ns |
| Are SDs significantly different (P < 0.05)? | No | No | No |
|  |  |  |  |
| **Data summary** |  |  |  |
| Number of treatments (columns) | 5 | 5 | 5 |
| Number of values (total) | 25 | 25 | 25 |

| **ANOVA table Prophenoloxidase** | **SS** | **DF** | **MS** | **F (DFn, DFd)** | **P value** |
| --- | --- | --- | --- | --- | --- |
| Treatment (between columns) | 0,2735 | 4 | 0,06837 | F (4, 20) = 46,50 | P<0,0001 |
| Residual (within columns) | 0,02941 | 20 | 0,001470 |  |  |
| Total | 0,3029 | 24 |  |  |  |
|  |  |  |  |  |  |
| **ANOVA table Lysozyme** | **SS** | **DF** | **MS** | **F (DFn, DFd)** | **P value** |
| Treatment (between columns) | 0,2382 | 4 | 0,05956 | F (4, 20) = 3,260 | P=0,0327 |
| Residual (within columns) | 0,3654 | 20 | 0,01827 |  |  |
| Total | 0,6036 | 24 |  |  |  |
|  |  |  |  |  |  |
| **ANOVA table Bactericidal capacity** | **SS** | **DF** | **MS** | **F (DFn, DFd)** | **P value** |
| Treatment (between columns) | 273,1 | 4 | 68,28 | F (4, 19) = 7,923 | P=0,0006 |
| Residual (within columns) | 163,7 | 19 | 8,618 |  |  |
| Total | 436,9 | 23 |  |  |  |

Supplementary Table S10.6. Summary of one-way ANOVA oxidative stress.

| **Table Analyzed** |  |  |  |  |  |
| --- | --- | --- | --- | --- | --- |
|  |  |  |  |  |  |
| **ANOVA summary** | **SOD** | **CAT** | **tGSH** | **PC** | **LPO** |
| F | 5,260 | 0,9284 | 34,63 | 0,5841 | 8,305 |
| P value | 0,0046 | 0,4673 | <0,0001 | 0,6778 | 0,0004 |
| P value summary | ** | ns | **** | ns | *** |
| Significant diff. among means (P < 0.05)? | Yes | No | Yes | No | Yes |
| R squared | 0,5127 | 0,1566 | 0,8738 | 0,1046 | 0,6242 |
|  |  |  |  |  |  |
| **Brown-Forsythe test** |  |  |  |  |  |
| F (DFn, DFd) | 0,2205 (4, 20) | 0,3720 (4, 20) | 0,3090 (4, 20) | 1,786 (4, 20) | 0,9123 (4, 20) |
| P value | 0,9238 | 0,8258 | 0,8685 | 0,1712 | 0,4759 |
| P value summary | ns | ns | ns | ns | ns |
| Are SDs significantly different (P < 0.05)? | No | No |  |  |  |
|  |  |  |  |  |  |
| **Data summary** |  |  |  |  |  |
| Number of treatments (columns) | 5 | 5 | 5 | 5 | 5 |
| Number of values (total) | 25 | 25 | 25 | 25 | 25 |

| **ANOVA table SOD** | **SS** | **DF** | **MS** | **F (DFn, DFd)** | **P value** |
| --- | --- | --- | --- | --- | --- |
| Treatment (between columns) | 128,0 | 4 | 31,99 | F (4, 20) = 5,260 | P=0,0046 |
| Residual (within columns) | 121,6 | 20 | 6,081 |  |  |
| Total | 249,6 | 24 |  |  |  |
|  |  |  |  |  |  |
| **ANOVA table CAT** | **SS** | **DF** | **MS** | **F (DFn, DFd)** | **P value** |
| Treatment (between columns) | 57,41 | 4 | 14,35 | F (4, 20) = 0,9284 | P=0,4673 |
| Residual (within columns) | 309,2 | 20 | 15,46 |  |  |
| Total | 366,6 | 24 |  |  |  |
|  |  |  |  |  |  |
| **ANOVA table tGSH** | **SS** | **DF** | **MS** | **F (DFn, DFd)** | **P value** |
| Treatment (between columns) | 524,9 | 4 | 131,2 | F (4, 20) = 34,63 | P<0,0001 |
| Residual (within columns) | 75,80 | 20 | 3,790 |  |  |
| Total | 600,7 | 24 |  |  |  |
|  |  |  |  |  |  |
| **ANOVA table PC** | **SS** | **DF** | **MS** | **F (DFn, DFd)** | **P value** |
| Treatment (between columns) | 17,90 | 4 | 4,476 | F (4, 20) = 0,5841 | P=0,6778 |
| Residual (within columns) | 153,3 | 20 | 7,664 |  |  |
| Total | 171,2 | 24 |  |  |  |
|  |  |  |  |  |  |
| **ANOVA table LPO** | **SS** | **DF** | **MS** | **F (DFn, DFd)** | **P value** |
| Treatment (between columns) | 337,6 | 4 | 84,40 | F (4, 20) = 8,305 | P=0,0004 |
| Residual (within columns) | 203,3 | 20 | 10,16 |  |  |
| Total | 540,9 | 24 |  |  |  |

Supplementary Table S10.7. Summary of one-way ANOVA aminotransferase activity.

| **Table Analyzed** |  |  |
| --- | --- | --- |
|  |  |  |
| **ANOVA summary** | **ALT activity** | **AST activity** |
| F | 27,01 | 11,30 |
| P value | <0,0001 | <0,0001 |
| P value summary | **** | **** |
| Significant diff. among means (P < 0.05)? | Yes | Yes |
| R squared | 0,8438 | 0,6933 |
|  |  |  |
| **Brown-Forsythe test** |  |  |
| F (DFn, DFd) | 0,6390 (4, 20) | 0,09883 (4, 20) |
| P value | 0,6407 | 0,9816 |
| P value summary | ns | ns |
| Are SDs significantly different (P < 0.05)? | No | No |
|  |  |  |
| **Data summary** |  |  |
| Number of treatments (columns) | 5 | 5 |
| Number of values (total) | 25 | 25 |

| **ANOVA table ALT** | **SS** | **DF** | **MS** | **F (DFn, DFd)** | **P value** |
| --- | --- | --- | --- | --- | --- |
| Treatment (between columns) | 120,0 | 4 | 30,00 | F (4, 20) = 27,01 | P<0,0001 |
| Residual (within columns) | 22,21 | 20 | 1,111 |  |  |
| Total | 142,2 | 24 |  |  |  |
|  |  |  |  |  |  |
| **ANOVA table AST** | **SS** | **DF** | **MS** | **F (DFn, DFd)** | **P value** |
| Treatment (between columns) | 673,9 | 4 | 168,5 | F (4, 20) = 11,30 | P<0,0001 |
| Residual (within columns) | 298,1 | 20 | 14,90 |  |  |
| Total | 972,0 | 24 |  |  |  |

Supplementary Table S10.8. Summary of one-way ANOVA free essential amino acids.

| **Table Analyzed** |  |  | |  | |  | |  | |  |
| --- | --- | --- | --- | --- | --- | --- | --- | --- | --- | --- |
|  |  |  | |  | |  | |  | |  |
| **ANOVA summary** | **Arginine** | **Histidine** | | **Isoleucine** | | **Leucine** | | **Lysine** | | **Threonine** |
| F | 72,30 | 11,56 | | 4,073 | | 12,56 | | 5,346 | | 54,44 |
| P value | <0,0001 | <0,0001 | | 0,0142 | | <0,0001 | | 0,0043 | | <0,0001 |
| P value summary | **** | **** | | * | | **** | | ** | | **** |
| Significant diff. among means (P < 0.05)? | Yes | Yes | | Yes | | Yes | | Yes | | Yes |
| R squared | 0,9353 | 0,6980 | | 0,4489 | | 0,7153 | | 0,5167 | | 0,9159 |
|  |  |  | |  | |  | |  | |  |
| **Brown-Forsythe test** |  |  | |  | |  | |  | |  |
| F (DFn, DFd) | 0,1220 (4, 20) | 0,7582 (4, 20) | | 0,6887 (4, 20) | | 0,5209 (4, 20) | | 0,8999 (4, 20) | | 0,3806 (4, 20) |
| P value | 0,9729 | 0,5645 | | 0,6082 | | 0,7215 | | 0,4826 | | 0,8198 |
| P value summary | ns | ns | | ns | | ns | | ns | | ns |
| Are SDs significantly different (P < 0.05)? | No | No | | No | | No | | No | | No |
|  |  |  | |  | |  | |  | |  |
| **Data summary** |  |  | |  | |  | |  | |  |
| Number of treatments (columns) | 5 | 5 | | 5 | | 5 | | 5 | | 5 |
| Number of values (total) | 25 | 25 | | 25 | | 25 | | 25 | | 25 |
|  |  |  | |  | |  | |  | |  |
| **Table Analyzed** |  |  | |  | |  | |  | |  |
|  |  |  | |  | |  | |  | |  |
| **ANOVA summary** | **Tryptophan** | **Valine** | **Methionine** | | **Cysteine** | | **Phenylalanine** | | **Tyrosine** | **Total** |
| F | 4,909 | 17,57 | 3,053 | | 20,27 | | 7,311 | | 332,5 | 10,29 |
| P value | 0,0064 | <0,0001 | 0,0408 | | <0,0001 | | 0,0008 | | <0,0001 | 0,0001 |
| P value summary | ** | **** | * | | **** | | *** | | **** | *** |
| Significant diff. among means (P < 0.05)? | Yes | Yes | Yes | | Yes | | Yes | | Yes | Yes |
| R squared | 0,4954 | 0,7784 | 0,3791 | | 0,8022 | | 0,5939 | | 0,9852 | 0,6730 |
|  |  |  |  | |  | |  | |  |  |
| **Brown-Forsythe test** |  |  |  | |  | |  | |  |  |
| F (DFn, DFd) | 0,1062 (4, 20) | 0,2567 (4, 20) | 0,9159 (4, 20) | | 0,1587 (4, 20) | | 0,9101 (4, 20) | | 2,321 (4, 20) | 0,5260 (4, 20) |
| P value | 0,9790 | 0,9022 | 0,4740 | | 0,9567 | | 0,4771 | | 0,0921 | 0,7179 |
| P value summary | ns | ns | ns | | ns | | ns | | ns | ns |
| Are SDs significantly different (P < 0.05)? | No | No | No | | No | | No | | No | No |
|  |  |  |  | |  | |  | |  |  |
| **Data summary** |  |  |  | |  | |  | |  |  |
| Number of treatments (columns) | 5 | 5 | 5 | | 5 | | 5 | | 5 | 5 |
| Number of values (total) | 25 | 25 | 25 | | 25 | | 25 | | 25 | 25 |

| **ANOVA table Arginine** | **SS** | **DF** | **MS** | **F (DFn, DFd)** | **P value** |
| --- | --- | --- | --- | --- | --- |
| Treatment (between columns) | 509,2 | 4 | 127,3 | F (4, 20) = 72,30 | P<0,0001 |
| Residual (within columns) | 35,21 | 20 | 1,761 |  |  |
| Total | 544,4 | 24 |  |  |  |
|  |  |  |  |  |  |
| **ANOVA table Histidine** | **SS** | **DF** | **MS** | **F (DFn, DFd)** | **P value** |
| Treatment (between columns) | 42,47 | 4 | 10,62 | F (4, 20) = 11,56 | P<0,0001 |
| Residual (within columns) | 18,37 | 20 | 0,9187 |  |  |
| Total | 60,84 | 24 |  |  |  |
|  |  |  |  |  |  |
| **ANOVA table Isoleucine** | **SS** | **DF** | **MS** | **F (DFn, DFd)** | **P value** |
| Treatment (between columns) | 6,858 | 4 | 1,714 | F (4, 20) = 4,073 | P=0,0142 |
| Residual (within columns) | 8,419 | 20 | 0,4210 |  |  |
| Total | 15,28 | 24 |  |  |  |
|  |  |  |  |  |  |
| **ANOVA table Leucine** | **SS** | **DF** | **MS** | **F (DFn, DFd)** | **P value** |
| Treatment (between columns) | 38,02 | 4 | 9,505 | F (4, 20) = 12,56 | P<0,0001 |
| Residual (within columns) | 15,13 | 20 | 0,7566 |  |  |
| Total | 53,15 | 24 |  |  |  |
|  |  |  |  |  |  |
| **ANOVA table Lysine** | **SS** | **DF** | **MS** | **F (DFn, DFd)** | **P value** |
| Treatment (between columns) | 3,862 | 4 | 0,9654 | F (4, 20) = 5,346 | P=0,0043 |
| Residual (within columns) | 3,611 | 20 | 0,1806 |  |  |
| Total | 7,473 | 24 |  |  |  |
|  |  |  |  |  |  |
| **ANOVA table Threonine** | **SS** | **DF** | **MS** | **F (DFn, DFd)** | **P value** |
| Treatment (between columns) | 195,8 | 4 | 48,96 | F (4, 20) = 54,44 | P<0,0001 |
| Residual (within columns) | 17,99 | 20 | 0,8993 |  |  |
| Total | 213,8 | 24 |  |  |  |
|  |  |  |  |  |  |
| **ANOVA table Tryptophan** | **SS** | **DF** | **MS** | **F (DFn, DFd)** | **P value** |
| Treatment (between columns) | 2,976 | 4 | 0,7440 | F (4, 20) = 4,909 | P=0,0064 |
| Residual (within columns) | 3,031 | 20 | 0,1516 |  |  |
| Total | 6,007 | 24 |  |  |  |
|  |  |  |  |  |  |
| **ANOVA table Valine** | **SS** | **DF** | **MS** | **F (DFn, DFd)** | **P value** |
| Treatment (between columns) | 21,54 | 4 | 5,386 | F (4, 20) = 17,57 | P<0,0001 |
| Residual (within columns) | 6,132 | 20 | 0,3066 |  |  |
| Total | 27,68 | 24 |  |  |  |
|  |  |  |  |  |  |
| **ANOVA table Methionine** | **SS** | **DF** | **MS** | **F (DFn, DFd)** | **P value** |
| Treatment (between columns) | 9,289 | 4 | 2,322 | F (4, 20) = 3,053 | P=0,0408 |
| Residual (within columns) | 15,21 | 20 | 0,7606 |  |  |
| Total | 24,50 | 24 |  |  |  |
|  |  |  |  |  |  |
| **ANOVA table Cysteine** | **SS** | **DF** | **MS** | **F (DFn, DFd)** | **P value** |
| Treatment (between columns) | 0,02774 | 4 | 0,006934 | F (4, 20) = 20,27 | P<0,0001 |
| Residual (within columns) | 0,006840 | 20 | 0,0003420 |  |  |
| Total | 0,03458 | 24 |  |  |  |
|  |  |  |  |  |  |
| **ANOVA table Phenylalanine** | **SS** | **DF** | **MS** | **F (DFn, DFd)** | **P value** |
| Treatment (between columns) | 28,37 | 4 | 7,091 | F (4, 20) = 7,311 | P=0,0008 |
| Residual (within columns) | 19,40 | 20 | 0,9700 |  |  |
| Total | 47,76 | 24 |  |  |  |
|  |  |  |  |  |  |
| **ANOVA table Tyrosine** | **SS** | **DF** | **MS** | **F (DFn, DFd)** | **P value** |
| Treatment (between columns) | 67,77 | 4 | 16,94 | F (4, 20) = 332,5 | P<0,0001 |
| Residual (within columns) | 1,019 | 20 | 0,05095 |  |  |
| Total | 68,78 | 24 |  |  |  |
|  |  |  |  |  |  |
| **ANOVA table Total** | **SS** | **DF** | **MS** | **F (DFn, DFd)** | **P value** |
| Treatment (between columns) | 548,5 | 4 | 137,1 | F (4, 20) = 10,29 | P=0,0001 |
| Residual (within columns) | 266,5 | 20 | 13,33 |  |  |
| Total | 815,1 | 24 |  |  |  |

Supplementary Table S10.9. Summary of one-way ANOVA free non-essential amino acids.

| **Table Analyzed** |  |  |  |  |  |  |  |
| --- | --- | --- | --- | --- | --- | --- | --- |
|  |  |  |  |  |  |  |  |
| **ANOVA summary** | **Aspartate** | **Glutamate** | **Alanine** | **Glycine** | **Proline** | **Serine** | **Total** |
| F | 1,401 | 83,94 | 63,17 | 37,33 | 18,91 | 15,95 | 77,14 |
| P value | 0,2696 | <0,0001 | <0,0001 | <0,0001 | <0,0001 | <0,0001 | <0,0001 |
| P value summary | ns | **** | **** | **** | **** | **** | **** |
| Significant diff. among means (P < 0.05)? | No | Yes | Yes | Yes | Yes | Yes | Yes |
| R squared | 0,2189 | 0,9438 | 0,9266 | 0,8819 | 0,7909 | 0,7613 | 0,9391 |
|  |  |  |  |  |  |  |  |
| **Brown-Forsythe test** |  |  |  |  |  |  |  |
| F (DFn, DFd) | 1,823 (4, 20) | 0,1244 (4, 20) | 0,3256 (4, 20) | 0,7064 (4, 20) | 0,7117 (4, 20) | 1,776 (4, 20) | 0,4458 (4, 20) |
| P value | 0,1640 | 0,9720 | 0,8574 | 0,5969 | 0,5935 | 0,1734 | 0,7741 |
| P value summary | ns | ns | ns | ns | ns | ns | ns |
| Are SDs significantly different (P < 0.05)? | No | No | No | No | No | No | No |
|  |  |  |  |  |  |  |  |
| **Data summary** |  |  |  |  |  |  |  |
| Number of treatments (columns) | 5 | 5 | 5 | 5 | 5 | 5 | 5 |
| Number of values (total) | 25 | 25 | 25 | 25 | 25 | 25 | 25 |

| **ANOVA table Aspartate** | **SS** | **DF** | **MS** | **F (DFn, DFd)** | **P value** |
| --- | --- | --- | --- | --- | --- |
| Treatment (between columns) | 1,737 | 4 | 0,4342 | F (4, 20) = 1,401 | P=0,2696 |
| Residual (within columns) | 6,198 | 20 | 0,3099 |  |  |
| Total | 7,935 | 24 |  |  |  |
|  |  |  |  |  |  |
| **ANOVA table Glutamate** | **SS** | **DF** | **MS** | **F (DFn, DFd)** | **P value** |
| Treatment (between columns) | 432,3 | 4 | 108,1 | F (4, 20) = 83,94 | P<0,0001 |
| Residual (within columns) | 25,75 | 20 | 1,287 |  |  |
| Total | 458,0 | 24 |  |  |  |
|  |  |  |  |  |  |
| **ANOVA table Alanine** | **SS** | **DF** | **MS** | **F (DFn, DFd)** | **P value** |
| Treatment (between columns) | 202,3 | 4 | 50,57 | F (4, 20) = 63,17 | P<0,0001 |
| Residual (within columns) | 16,01 | 20 | 0,8005 |  |  |
| Total | 218,3 | 24 |  |  |  |
|  |  |  |  |  |  |
| **ANOVA table Glycine** | **SS** | **DF** | **MS** | **F (DFn, DFd)** | **P value** |
| Treatment (between columns) | 189,2 | 4 | 47,31 | F (4, 20) = 37,33 | P<0,0001 |
| Residual (within columns) | 25,35 | 20 | 1,267 |  |  |
| Total | 214,6 | 24 |  |  |  |
|  |  |  |  |  |  |
| **ANOVA table Proline** | **SS** | **DF** | **MS** | **F (DFn, DFd)** | **P value** |
| Treatment (between columns) | 87,35 | 4 | 21,84 | F (4, 20) = 18,91 | P<0,0001 |
| Residual (within columns) | 23,10 | 20 | 1,155 |  |  |
| Total | 110,5 | 24 |  |  |  |
| **ANOVA table Serine** | **SS** | **DF** | **MS** | **F (DFn, DFd)** | **P value** |
| Treatment (between columns) | 31,13 | 4 | 7,783 | F (4, 20) = 15,95 | P<0,0001 |
| Residual (within columns) | 9,760 | 20 | 0,4880 |  |  |
| Total | 40,89 | 24 |  |  |  |
|  |  |  |  |  |  |
| **ANOVA table Total** | **SS** | **DF** | **MS** | **F (DFn, DFd)** | **P value** |
| Treatment (between columns) | 2429 | 4 | 607,3 | F (4, 20) = 77,14 | P<0,0001 |
| Residual (within columns) | 157,5 | 20 | 7,873 |  |  |
| Total | 2587 | 24 |  |  |  |

Supplementary Table S10.10. Summary of one-way ANOVA nitrogen metabolites.

| **Table Analyzed** |  |  |  |  |  |  |  |
| --- | --- | --- | --- | --- | --- | --- | --- |
|  |  |  |  |  |  |  |  |
| **ANOVA summary** | **Asparagine** | **Glutamine** | **Taurine** | **Ornithine** | **GABA** | **Hydroxyproline** |  |
| F | 108,4 | 2,982 | 27,83 | 47,36 | 51,09 | 62,80 |  |
| P value | <0,0001 | 0,0441 | <0,0001 | <0,0001 | <0,0001 | <0,0001 |  |
| P value summary | **** | * | **** | **** | **** | **** |  |
| Significant diff. among means (P < 0.05)? | Yes | Yes | Yes | Yes | Yes | Yes |  |
| R squared | 0,9559 | 0,3736 | 0,8477 | 0,9045 | 0,9109 | 0,9263 |  |
|  |  |  |  |  |  |  |  |
| **Brown-Forsythe test** |  |  |  |  |  |  |  |
| F (DFn, DFd) | 1,873 (4, 20) | 0,9794 (4, 20) | 2,032 (4, 20) | 1,850 (4, 20) | 0,6667 (4, 20) | 1,462 (4, 20) |  |
| P value | 0,1547 | 0,4410 | 0,1284 | 0,1589 | 0,6226 | 0,2510 |  |
| P value summary | ns | ns | ns | ns | ns | ns |  |
| Are SDs significantly different (P < 0.05)? | No | No | No | No | No | No |  |
|  |  |  |  |  |  |  |  |
| **Data summary** |  |  |  |  |  |  |  |
| Number of treatments (columns) | 5 | 5 | 5 | 5 | 5 | 5 |  |
| Number of values (total) | 25 | 25 | 25 | 25 | 25 | 25 |  |
|  |  |  |  |  |  |  |  |
| **Table Analyzed** |  |  |  |  |  |  |  |
|  |  |  |  |  |  |  |  |
| **ANOVA summary** | **B-alanine** | **Homocysteine** | **Cystathione** | **Trimethylglycine** | **S-adenosylmet.** | **S-adenosylhomocys.** | **Total** |
| F | 9,266 | 1,669 | 79,40 | 185,7 | 42,25 | 1,819 | 12,62 |
| P value | 0,0002 | 0,1966 | <0,0001 | <0,0001 | <0,0001 | 0,1647 | <0,0001 |
| P value summary | *** | ns | **** | **** | **** | ns | **** |
| Significant diff. among means (P < 0.05)? | Yes | No | Yes | Yes | Yes | No | Yes |
| R squared | 0,6495 | 0,2502 | 0,9408 | 0,9738 | 0,8942 | 0,2668 | 0,7163 |
|  |  |  |  |  |  |  |  |
| **Brown-Forsythe test** |  |  |  |  |  |  |  |
| F (DFn, DFd) | 1,097 (4, 20) | 1,532 (4, 20) | 1,786 (4, 20) | 1,541 (4, 20) | 1,222 (4, 20) | 2,370 (4, 20) | 1,241 (4, 20) |
| P value | 0,3852 | 0,2309 | 0,1712 | 0,2286 | 0,3330 | 0,0871 | 0,3254 |
| P value summary | ns | ns | ns | ns | ns | ns | ns |
| Are SDs significantly different (P < 0.05)? | No | No | No | No | No | No | No |
|  |  |  |  |  |  |  |  |
| **Data summary** |  |  |  |  |  |  |  |
| Number of treatments (columns) | 5 | 5 | 5 | 5 | 5 | 5 | 5 |
| Number of values (total) | 25 | 25 | 25 | 25 | 25 | 25 | 25 |

| **ANOVA table Asparagine** | **SS** | **DF** | **MS** | **F (DFn, DFd)** | **P value** |
| --- | --- | --- | --- | --- | --- |
| Treatment (between columns) | 13,24 | 4 | 3,310 | F (4, 20) = 108,4 | P<0,0001 |
| Residual (within columns) | 0,6107 | 20 | 0,03054 |  |  |
| Total | 13,85 | 24 |  |  |  |
|  |  |  |  |  |  |
| **ANOVA table Glutamine** | **SS** | **DF** | **MS** | **F (DFn, DFd)** | **P value** |
| Treatment (between columns) | 19,35 | 4 | 4,839 | F (4, 20) = 2,982 | P=0,0441 |
| Residual (within columns) | 32,46 | 20 | 1,623 |  |  |
| Total | 51,81 | 24 |  |  |  |
|  |  |  |  |  |  |
| **ANOVA table Taurine** | **SS** | **DF** | **MS** | **F (DFn, DFd)** | **P value** |
| Treatment (between columns) | 66,41 | 4 | 16,60 | F (4, 20) = 27,83 | P<0,0001 |
| Residual (within columns) | 11,93 | 20 | 0,5965 |  |  |
| Total | 78,34 | 24 |  |  |  |
|  |  |  |  |  |  |
| **ANOVA table Ornithine** | **SS** | **DF** | **MS** | **F (DFn, DFd)** | **P value** |
| Treatment (between columns) | 2,313 | 4 | 0,5782 | F (4, 20) = 47,36 | P<0,0001 |
| Residual (within columns) | 0,2442 | 20 | 0,01221 |  |  |
| Total | 2,557 | 24 |  |  |  |
|  |  |  |  |  |  |
| **ANOVA table GABA** | **SS** | **DF** | **MS** | **F (DFn, DFd)** | **P value** |
| Treatment (between columns) | 0,3609 | 4 | 0,09022 | F (4, 20) = 51,09 | P<0,0001 |
| Residual (within columns) | 0,03532 | 20 | 0,001766 |  |  |
| Total | 0,3962 | 24 |  |  |  |
|  |  |  |  |  |  |
| **ANOVA table Hydroxyproline** | **SS** | **DF** | **MS** | **F (DFn, DFd)** | **P value** |
| Treatment (between columns) | 0,2924 | 4 | 0,07310 | F (4, 20) = 62,80 | P<0,0001 |
| Residual (within columns) | 0,02328 | 20 | 0,001164 |  |  |
| Total | 0,3157 | 24 |  |  |  |
|  |  |  |  |  |  |
| **ANOVA table B-alanine** |  |  |  |  |  |
| Treatment (between columns) | 0,01312 | 4 | 0,003280 | F (4, 20) = 9,266 | P=0,0002 |
| Residual (within columns) | 0,007080 | 20 | 0,0003540 |  |  |
| Total | 0,02020 | 24 |  |  |  |
|  |  |  |  |  |  |
| **ANOVA table Homocysteine** | **SS** | **DF** | **MS** | **F (DFn, DFd)** | **P value** |
| Treatment (between columns) | 6,930e-006 | 4 | 1,733e-006 | F (4, 20) = 1,669 | P=0,1966 |
| Residual (within columns) | 2,076e-005 | 20 | 1,038e-006 |  |  |
| Total | 2,769e-005 | 24 |  |  |  |
|  |  |  |  |  |  |
| **ANOVA table Cystathione** | **SS** | **DF** | **MS** | **F (DFn, DFd)** | **P value** |
| Treatment (between columns) | 0,002612 | 4 | 0,0006531 | F (4, 20) = 79,40 | P<0,0001 |
| Residual (within columns) | 0,0001645 | 20 | 8,225e-006 |  |  |
| Total | 0,002777 | 24 |  |  |  |
|  |  |  |  |  |  |
| **ANOVA table Trimethylglycine** | **SS** | **DF** | **MS** | **F (DFn, DFd)** | **P value** |
| Treatment (between columns) | 0,004602 | 4 | 0,001151 | F (4, 20) = 185,7 | P<0,0001 |
| Residual (within columns) | 0,0001239 | 20 | 6,197e-006 |  |  |
| Total | 0,004726 | 24 |  |  |  |
|  |  |  |  |  |  |
| **ANOVA table S-adenosylmethionine** | **SS** | **DF** | **MS** | **F (DFn, DFd)** | **P value** |
| Treatment (between columns) | 0,3630 | 4 | 0,09076 | F (4, 20) = 42,25 | P<0,0001 |
| Residual (within columns) | 0,04296 | 20 | 0,002148 |  |  |
| Total | 0,4060 | 24 |  |  |  |
|  |  |  |  |  |  |
| **ANOVA table S-adenosylhomocysteine** | **SS** | **DF** | **MS** | **F (DFn, DFd)** | **P value** |
| Treatment (between columns) | 0,002896 | 4 | 0,0007240 | F (4, 20) = 1,819 | P=0,1647 |
| Residual (within columns) | 0,007960 | 20 | 0,0003980 |  |  |
| Total | 0,01086 | 24 |  |  |  |
|  |  |  |  |  |  |
| **ANOVA table Total** | **SS** | **DF** | **MS** | **F (DFn, DFd)** | **P value** |
| Treatment (between columns) | 121,4 | 4 | 30,35 | F (4, 20) = 12,62 | P<0,0001 |
| Residual (within columns) | 48,09 | 20 | 2,404 |  |  |
| Total | 169,5 | 24 |  |  |  |
